# Signal termination of the chemokine receptor CCR9 is governed by an arrestin-independent phosphorylation mechanism

**DOI:** 10.1016/j.jbc.2025.108462

**Published:** 2025-03-26

**Authors:** Thomas D. Lamme, Martine J. Smit, Christopher T. Schafer

**Affiliations:** Faculty of Science, Division of Medicinal Chemistry, Department of Chemistry and Pharmaceutical Sciences, Amsterdam Institute for Molecular and Life Sciences (AIMMS), Vrije Universiteit Amsterdam, Amsterdam, The Netherlands

**Keywords:** G protein–coupled receptor, phosphorylation, chemokine, G protein, chemokine receptor

## Abstract

The C–C chemokine receptor type 9 (CCR9) coordinates immune cell migration from the thymus to the small intestine along gradients of the chemokine CCL25. Receptor dysregulation is associated with a variety of inflammatory bowel diseases such as Crohn's and ulcerative colitis, whereas aberrant CCR9 overexpression correlates with tumor metastasis. Despite being an attractive therapeutic target, attempts to clinically antagonize CCR9 have been unsuccessful. This highlights the need for a deeper understanding of its specific regulatory mechanisms and signaling pathways. CCR9 is a G protein–coupled receptor (GPCR) and activates G_i_ and G_q_ pathways. Unexpectedly, live-cell bioluminescence resonance energy transfer assays reveal only limited G protein activation, and signaling is rapidly terminated. Truncating the receptor C terminus significantly enhanced G protein coupling, highlighting a regulatory role of this domain. Signal suppression was not because of canonical arrestin-coordinated desensitization. Rather, removal of GPCR kinase phosphorylation led to sustained and robust G protein activation by CCR9. Using site-directed mutagenesis, we identified specific phosphorylation motifs that attenuate G protein coupling. Receptor internalization did not correlate with G protein activation capabilities. Instead, CCR9 phosphorylation disrupted the interaction of G protein heterotrimers with the receptor. This interference may lead to rapid loss of productive coupling and downstream signaling as phosphorylation would effectively render the receptor incapable of G protein coupling. An arrestin-independent, phosphorylation-driven deactivation mechanism could complement arrestin-dependent regulation of other GPCRs and have consequences for therapeutically targeting these receptors.

Chemokine receptors are class A G protein–coupled receptors (GPCRs) that drive cell migration during immune homeostasis, inflammation, and development in response to small protein agonists called chemokines. Binding of chemokines activates the receptors, inducing key conformational changes that promote G protein heterotrimer coupling, which activates the G protein and leads to guanyl nucleotide exchange within the Gα subunit and separation from the Gβγ dimer. Chemokine-directed cell migration is driven by signaling cascades initiated by the dissociated, active G protein. The activated receptors also recruit GPCR kinases (GRKs), which initiate signal termination by phosphorylating serine and threonine residues on the receptor cytoplasmic face and C terminus. These modifications lead to arrestin recruitment, which canonically blocks further receptor signaling by sterically occluding the G protein coupling interface. Arrestins also coordinate receptor internalization from the plasma membrane, where the GPCRs are either degraded or recycled for another round of signaling. Together, these effectors rapidly translate stimulation into larger cellular effects, while regulating signaling and overactivation (1, 2).

The C–C chemokine receptor type 9 (CCR9) mediates T-cell maturation and migration from the thymus to the small intestine along gradients of its sole endogenous chemokine ligand CCL25 (3, 4). Upon activation, the receptor couples to G_i_ and G_q_ proteins, which initiate signaling cascades that mediate migratory responses. The specifics of CCR9 signal regulation are unknown; however, the activated receptors recruit arrestins and show desensitization following overstimulation (5). Thus, signal termination can be assumed to follow the classical GRK to arrestin coordination described for GPCRs.

A key role of CCR9 is coordinating immune cell localization and positioning in the small intestine, which contributes to immune homeostasis and inflammatory responses. Receptor dysregulation is associated with a variety of inflammatory bowel diseases, as well as cardiovascular disease, arthritis, and others (6, 7, 8). In addition, CCR9 and CCL25 overexpression has been observed in malignant tumors and is correlated with metastasis to the colon (9, 10). Therefore, CCR9 has emerged as a potential therapeutic target for various diseases (7, 11, 12). Antagonizing CCR9 by small molecules effectively decreased inflammation in the colon in mouse models (13, 14); however, this effect was not replicated in clinical trials (15). Pharmacological inhibition of the receptor by specific monoclonal antibodies effectively inhibited T lymphoblastic leukemia tumor growth in preclinical studies (16, 17). Therefore, addressing CCR9-related pathophysiology may require more specific approaches, and further elucidating native receptor regulation may provide insights into alternative strategies.

Here, we present a detailed investigation of the signaling and regulation of CCR9 by the classical GPCR mediators, including G proteins, GRKs, and β-arrestins. Our results reveal CCR9 activation results in limited G protein signaling that is rapidly terminated. Rather than arrestins, GRK phosphorylation of specific C-terminal serine and threonine residues mediates the loss of G protein coupling. Phosphorylation appears to directly interfere with productive G protein coupling. We propose that the activation of CCR9 is tightly regulated by GRK phosphorylation *via* an arrestin-independent mechanism.

## Results

### CCR9 robustly recruits mini-G_i_ but shows limited activation of the full heterotrimer

**Figure 1:**
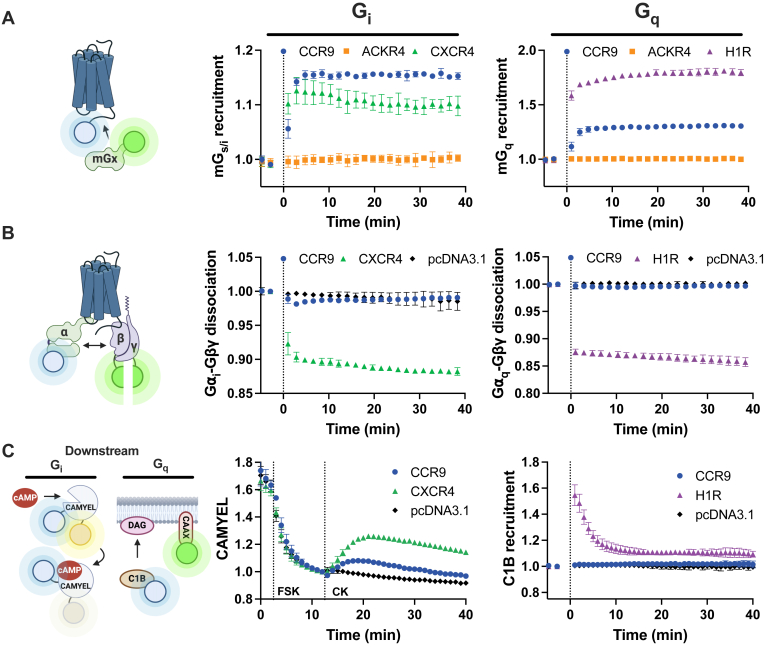
**CCR9 recruits G_i_ and G_q_ proteins but shows limited heterotrimer activation.***A,* ligand-induced recruitment of mini-G⍺_i_ (*left*) and mini-G⍺_q_ (*right*) to CCR9-RlucII, ACKR4-RlucII, and CXCR4-RlucII or H1R-RlucII in HEK293 cells measured by BRET following stimulation with 100 nM CCL25, CXCL12 or 10 μM histamine. *B,* ligand-induced activation of G_i_ and G_q_ proteins measured as dissociation of G⍺_i_-Nluc/G⍺_q_-Nluc and Gβγ-split-mVenus (Gβγ-smV) in HEK293 cells by CCR9 and CXCR4 or H1R upon stimulation of 100 nM CCL25, CXCL12, or 1 μM histamine, respectively. *C,* inhibition of cAMP production by CCR9 and CXCR4 in HEK293T using the BRET-based cAMP sensor CAMYEL (*left*). Cells were treated with 10 μM forskolin (FSK) to stimulate cAMP production for 10 min, followed by 100 nM chemokine (CK, CCL25, or CXCL12). Recruitment of C1B to the membrane in response of DAG production was measured by bystander BRET between Nluc-C1B and mV-CAAX (*right*) in HEK293T by CCR9 and H1R following stimulation with 100 nM CCL25 or 10 μM histamine, respectively. Values represent the mean ± SD of three independent experiments performed in triplicate. BRET, bioluminescence resonance energy transfer; CCR9, C–C chemokine receptor type 9; DAG, diacylglycerol; HEK293, human embryonic kidney 293T cell line; H1R, histamine H1 receptor.

Activation of CCR9 drives immune cell migration through the activation of G_i_ and G_q_ signaling pathways (18, 19, 20, 21, 22, 23, 24). G protein coupling can be generally described as several sequential and discrete steps, which we have simplified into three critical events. First, the G protein engages the active receptor, then G protein activation leads to separation of the Gα from the Gβγ subunits, and finally the active G protein coordinates downstream signaling responses. Initial recruitment was tested by tracking bioluminescence resonance energy transfer (BRET) between CCR9 with a C-terminal luciferase (CCR9-RlucII) and a fluorophore fused to a mini-G protein (mG_x_, where x refers to the Gα subtype being mimicked) (25). Mini-G proteins are engineered G protein mimetics containing the Ras domain of Gα_s_ and with the specific interacting residues of G_i_ or G_q_. When CCR9 is activated by CCL25, it leads to a rapid and robust recruitment of mG_i_ to the receptor with similar kinetics as observed for CXCR4, a well-studied and efficient activator of G_i_ proteins, stimulated by CXCL12 (Fig. 1*A*). Likewise, effective recruitment of mG_q_ is observed for CCR9 with analogous kinetics to the control G_q_-coupled histamine H1 receptor (H1R) upon histamine stimulation. In stark contrast, ACKR4, the other native CCL25-binding receptor, shows no change in BRET between the receptor and mG_i_ and mG_q_, consistent with the atypical nature of its activation and lack of G protein coupling (26).

Following recruitment, the G protein is activated, which leads to a structural rearrangement and dissociation of the Gα and Gβγ subunits. By introducing BRET sensors into the heterotrimer between the Gα and Gβγ subunits, G protein activation can be tracked by a decrease in signal following activation by a GPCR (27). Despite showing robust mG_i_ recruitment (Fig. 1*A*), stimulation of CCR9 by CCL25 only produces a modest decrease in BRET between the G protein subunits (ΔBRET ∼2%) that returns to basal levels within 10 min (Fig. 1*B*). CXCR4, serving here as a positive control, produces a proportionally large signal (ΔBRET ∼10%), which is sustained for the duration of the experiment. A similarly muted response to CCR9 activation is observed with G_q_ activation (Fig. 1*B*) suggesting this weak activation, despite strong recruitment, is a general feature of CCR9 signaling and not specific to certain G proteins. Increasing the amount of transfected CCR9, DNA only modestly increased G⍺_i_–Gβγ dissociation and was overshadowed by the CXCR4 response at all tested concentrations (Fig. S1).

The limited G protein activation is further observed in downstream signaling by G_i_ proteins. G_i_ activation inhibits cAMP production. To characterize this response, the inhibition of forskolin (FSK)-induced cAMP production by activated chemokine receptor was tracked using a BRET-based cAMP biosensor (CAMYEL) (28). Like with the G protein dissociation, CCR9 activation by CCL25 had a limited effect on cAMP levels compared with the mock-transfected cells, whereas CXCR4 activated by CXCL12 substantially reversed FSK-promoted cAMP production (Fig. 1*C*). A similar pattern was observed with downstream G_q_ signaling. G_q_ activation induces the formation of diacylglycerol (DAG) by phospholipase C β activation, which can be measured by the recruitment of the DAG-binding domain of protein kinase C δ (Nluc-C1B) toward the plasma membrane (mV-CAAX) (29). CCR9 activation did not increase C1B recruitment, whereas H1R stimulation by histamine induced a substantial effect. Due to a clearer response window, downstream responses (CAMYEL, C1B recruitment) are presented in human embryonic kidney 293T (HEK293T) cells. Comparable effects were observed in HEK293 cells (Fig. S2).

### The CCR9 C terminus suppresses CCL25-induced G protein coupling

**Figure 2:**
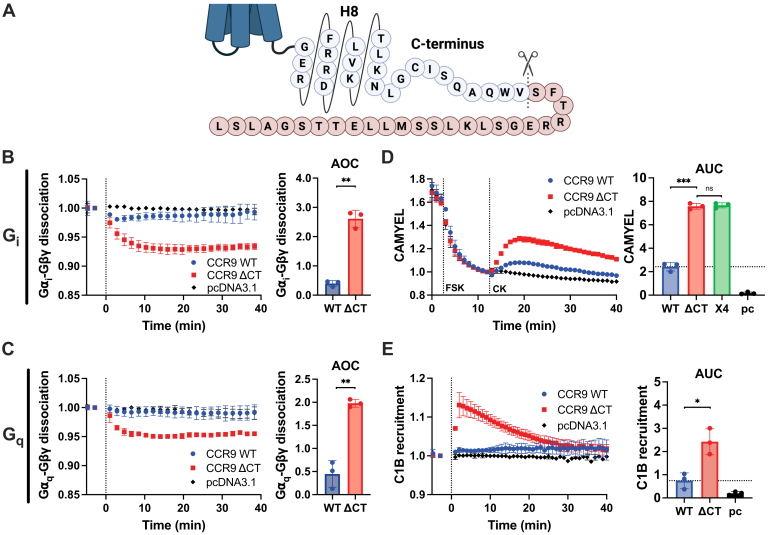
**Truncating the CCR9 C terminus significantly improves CCL25-induced G_i_ and G_q_ coupling.***A,* schematic view of the C-terminal truncation after V344 to produce CCR9 ΔCT. *B,* ligand-induced activation of G_i_ proteins measured as loss of G⍺_i_-Nluc and Gβγ-smV BRET in HEK293 cells by CCR9 and CCR9 ΔCT upon stimulation of 100 nM CCL25 (*left*). Quantification of G⍺_i_–Gβγ dissociation by integration of the area over the BRET curves (*right*). *C,* activation of G_q_ proteins following stimulation of CCR9 or CCR9 ΔCT with 100 nM CCL25 was measured by the dissociation of G⍺_q_-Nluc and Gβγ-smV in HEK293 cells (*left*) and quantified as AOC (*right*). *D,* inhibition of cAMP production was tracked by BRET using a CAMYEL sensor. Cells expressing either CCR9 or CCR9 ΔCT were stimulated by 10 μM FSK followed by 100 nM CCL25 (*left*) and quantified as the area under the curve (AUC) after chemokine addition (*right*). The data used for the CXCR4 analysis are presented in Fig. 1. *E,* DAG production following G_q_ activation was measured by bystander BRET between Nluc-C1B and mV-CAAX following activation of CCR9 or CCR9 ΔCT by 100 nM CCL25 (*left*). The data were quantified by AUC analysis following ligand addition (*right*). Values represent the mean ± SD of three independent experiments performed in triplicate. Statistical significance was determined by one-way Brown–Forsythe and Welch ANOVA followed by a Dunnett's T3 multiple comparisons test. ∗*p* < 0.05, ∗∗*p* < 0.001, and ∗∗∗*p* < 0.0001. AOC, area over the dissociation curve; CCR9, C–C chemokine receptor type 9. BRET, bioluminescence resonance energy transfer; DAG, diacylglycerol; FSK, forskolin; HEK293, human embryonic kidney 293 cell line.

The rapid CCR9 signal attenuation suggests that a fast inhibitory mechanism suppresses the coupling of G proteins to the active receptor. The GPCR C terminus plays a critical role in canonical signal termination by coordinating arrestin interactions following GRK phosphorylation and has recently been described in some cases as autoinhibitory (30, 31, 32) and therefore may contribute to the poor G protein activation by CCR9. To resolve whether the C terminus plays a role in regulation of CCR9 signaling, the receptor was truncated after position V334 (CCR9 ΔCT, Fig. 2*A*). Consistent with an inhibitory function, CCR9 ΔCT showed robust and sustained G_i_ and G_q_ protein dissociation following CCL25 stimulation (Fig. 2, *B* and *C*). The truncated receptor shows a greater maximal change in BRET (G_i_: ΔBRET ∼7% *versus* ∼2%, G_q_ ΔBRET ∼5% *versus* ∼0%) and a sixfold increase in G_i_ activation and fourfold for G_q_ as calculated by the area over the dissociation curve (AOC) (Fig. 2, *B* and *C*). Downstream signaling through G_i_ and G_q_ pathways, cAMP inhibition, and DAG production were significantly enhanced by CCR9 ΔCT compared with the WT receptor (Fig. 2, *D* and *E*). Inhibition of cAMP production by CCR9 ΔCT reached similar levels as CXCR4 (Fig. 2*D*), supporting the interpretation that the observed suppression is mediated by the CCR9 C terminus. The surface expression of CCR9 ΔCT was slightly reduced compared with the WT receptor (Fig. S3), suggesting that the signaling differences between CCR9 and CXCR4 are not because of different expression levels and emphasizing the gain of function with the tail truncation. Unlike other receptors (30, 32, 33), only the agonist-promoted G protein activation is enhanced for CCR9 ΔCT and constitutive activity is not altered (Fig. S4). These results suggest that the limited G protein activation by CCR9 is due to features of the receptor C terminus suppressing signaling by the activated receptor.

### Arrestins do not attenuate CCR9-mediated G protein signaling

**Figure 3:**
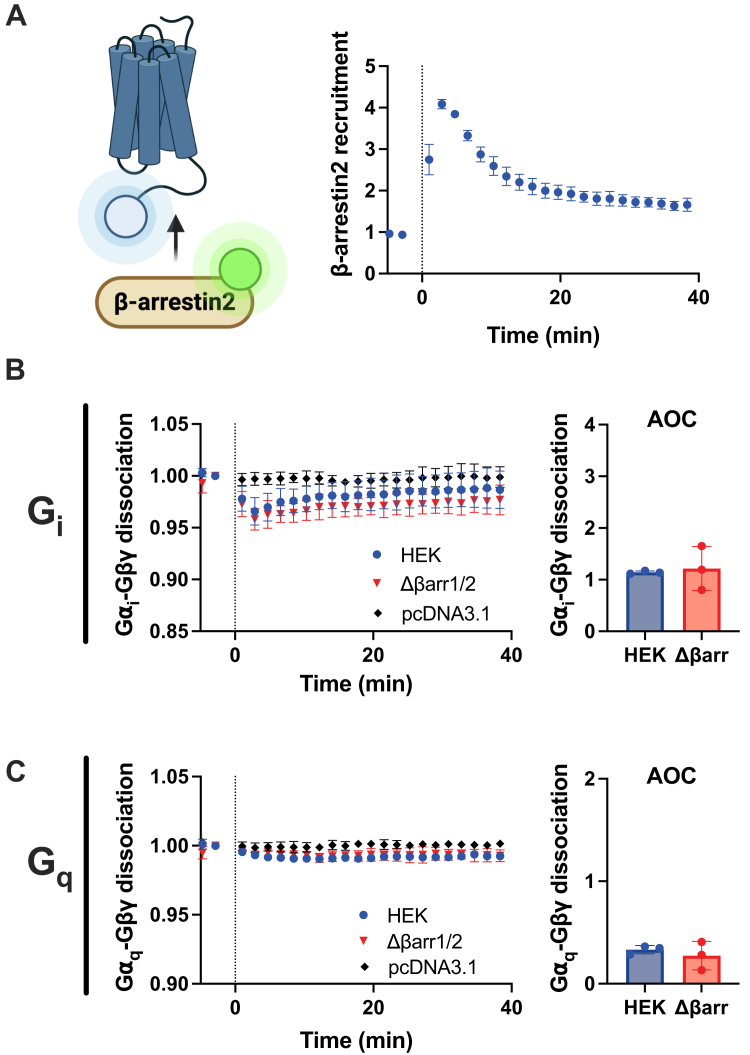
**Arrestins do not impact CCR9 coupling to G proteins.***A,* CCL25-induced recruitment of GFP10-βarr2 toward CCR9-RlucII detected by BRET following stimulation with 200 nM chemokine. G protein activation by CCR9 following 100 nM CCL25 stimulation was measured by BRET decrease between (*B*) Gα_i_-Nluc or (*C*) Gα_q_-Nluc and Gβγ-smV in HEK293 parental and HEK293 Δβarr1/2 cells (*left*). The traces were quantified by AOC analysis (*right*). Values represent the mean ± SD of three independent experiments performed in triplicate. AOC, area over the dissociation curve; BRET, bioluminescence resonance energy transfer; CCR9, C–C chemokine receptor type 9; HEK293, human embryonic kidney 293 cell line.

GPCR C termini canonically coordinate signal termination by acting as a scaffold for GRK phosphorylation, which in turn promotes arrestin recruitment to impede further G protein coupling (34, 35). As the CCR9 C terminus appears to be critical for the rapid suppression of signaling, we evaluated how arrestins influence the coupling of G proteins to CCR9 by tracking the activation of G proteins through G protein dissociation in β-arrestin1/2 CRISPR KO cells (Δβarr1/2) (36). Prior to evaluating the impact of arrestins on G proteins, we first assessed β-arrestin2 recruitment to CCL25-stimulated CCR9 by BRET between CCR9-RlucII and GFP-tagged arrestin (GFP10-βarr2). Agonist addition leads to a rapid association of arrestin to CCR9 that peaks at ∼4 min and then slowly decreases to ∼50% of the peak signal (Fig. 3*A*). Both G_i_ and G_q_ protein dissociation following CCL25 stimulation of CCR9 in Δβarr1/2 cells shows identical levels of activation as in WT cells and do not match the enhanced activation with CCR9 ΔCT (Figs. 3, *B and C*, *versus* 2, *B and C*). No change in constitutive activity was observed for CCR9 in Δβarr1/2 cells (Fig. S6*A*). These data suggest that the C-terminal suppression of CCR9 signaling is not because of arrestin coordination.

### Phosphorylation of CCR9 by GRKs suppresses G protein activation

**Figure 4:**
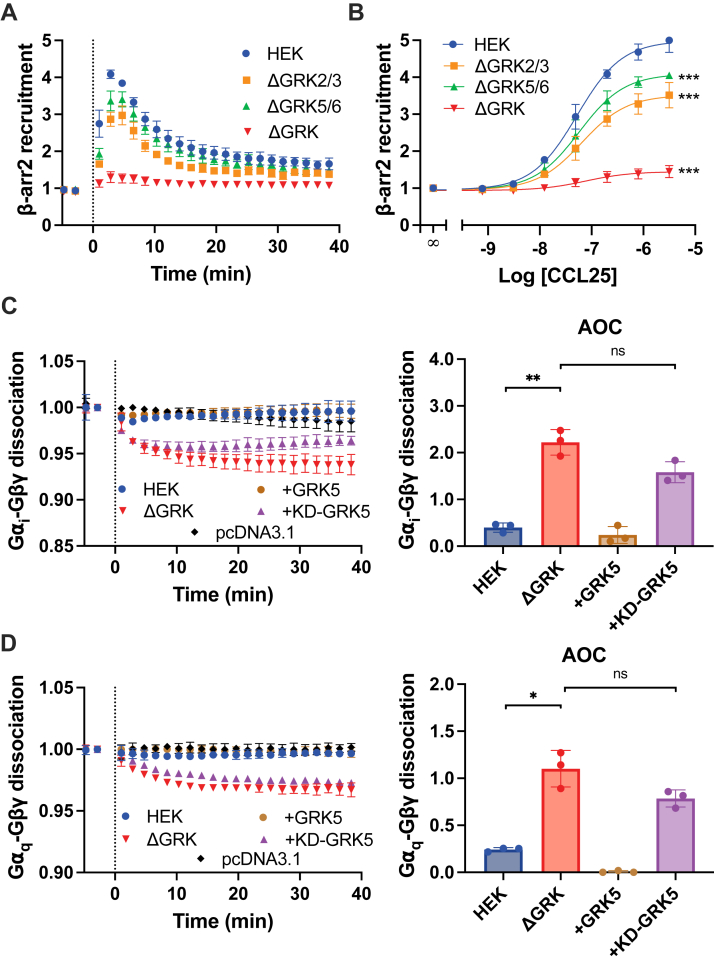
**G protein heterotrimer dissociation by CCR9 is enhanced in the absence of GRKs.***A,* recruitment of GFP10-βarr2 to CCR9-RlucII was tracked by BRET following stimulation with 200 nM CCL25 and (*B*) across a titration of CCL25 concentrations in HEK293, ΔGRK2/3, ΔGRK5/6, and ΔGRK2/3/5/6 (ΔGRK) cells. *C* and *D,* activation of G proteins by CCR9 stimulated with 100 nM CCL25 measured by the decrease in BRET between (*C*) Gα_i_-Nluc or (*D*) Gα_q_-Nluc and Gβγ-smV in HEK293 and ΔGRK cells (*left*). WT data were repeated from Fig. 1*B* for comparison. GRK5 or kinase dead GRK5 (KD-GRK5) were cotransfected as indicated. The traces were quantified by AOC analysis (*right*). Values represent the mean ± SD of three independent experiments performed in triplicate. Statistical significance was determined by one-way Brown–Forsythe and Welch ANOVA followed by a Dunnett's T3 multiple comparisons test (AOC) or using the extra sum-of-squares *F* test (DRC). ∗*p* < 0.05, ∗∗*p* < 0.001, and ∗∗∗*p* < 0.0001. AOC, area over the dissociation curve; BRET, bioluminescence resonance energy transfer; CCR9, C–C chemokine receptor type 9; DRC, dose–response curve; GRK, G protein–coupled receptor kinase; HEK293, human embryonic kidney 293 cell line.

In addition to coordinating arrestin recruitment, the GPCR C terminus can undergo phosphorylation at serine and threonine residues by GRKs, which drive arrestin-independent internalization in some cases (37, 38, 39). Therefore, GRKs can influence GPCR activity *via* modification and may coordinate the C-terminal suppression of CCR9. To determine the contributions of the four ubiquitously expressed GRKs (GRK2, 3, 5, and 6) to CCR9 phosphorylation, the recruitment of β-arrestin2 to CCR9 was measured in GRK-family CRISPR KO cells (ΔGRK2/3, ΔGRK5/6, and ΔGRK2/3/5/6) by BRET between the receptor and arrestin (Fig. 4, *A* and *B*) (40). Arrestins require phosphorylation of the GPCR C terminus for recruitment, and these interactions are sensitive to specific phosphorylation patterns. Thus, changes to arrestin binding correspond to changes in the receptor phosphorylation state and pattern. Arrestin recruitment is only partially impacted in ΔGRK2/3 or ΔGRK5/6 cells, suggesting little preference for CCR9 phosphorylation between the major GRK families. In ΔGRK2/3/5/6 cells, the recruitment is nearly eliminated. G protein activation by CCR9 in the ΔGRK2/3/5/6 cells, tracked by BRET through heterotrimer dissociation, revealed robust and sustained dissociation (Fig. 4, *C* and *D*) with a nearly identical profile to CCR9 ΔCT (Fig. 2, *B* and *C*). The ∼six-fold increase for G_i_ and the ∼four-fold increase for G_q_ in the AOC by CCR9-mediated activation in the absence of GRKs matches the increase for the CCR9 ΔCT mutant (Fig. 4, *C* and *D* and Fig. 2, *B* and *C*), suggesting that C-terminal suppression can be specifically attributed to GRKs, rather than other second-messenger–dependent protein kinases (41). No change in constitutive activity was observed for CCR9 in ΔGRK cells (Fig. S6*B*). Moreover, CCR9 activation did not show enhanced heterotrimer dissociation in ΔGRK2/3 and ΔGRK5/6 cells, indicating that the presence of any GRK efficiently suppresses G protein activation (Fig. S7). In addition, the surface expression of CCR9 is not altered in any of the KO-cell lines, confirming the enhanced activity is due to GRKs and not more receptors (Fig. S7*E*).

Although the canonical function of GRKs is to phosphorylate GPCR C termini, new evidence suggests the kinases can also serve as scaffold for complex formation (42). Thus, inhibition of CCR9 by GRKs may be due to phosphorylation but could also be due to noncanonical interactions between the effector and the receptor. To differentiate between these possibilities, G protein activation by CCR9 was tracked in ΔGRK2/3/5/6 cells cotransfected with WT GRK5 or a kinase-deficient GRK5 variant (K215R GRK5 = KD-GRK5) (40). Overexpression of GRK5 completely eliminated the G_i_ and G_q_ heterotrimer dissociation (Fig. 4, *C* and *D*). In contrast, expression of KD-GRK5 had no appreciable effect on the improved G protein activation in the absence of GRKs. Therefore, the contribution of GRKs to CCR9 signal termination is due to phosphorylation of the receptor and not an alternate interaction.

### Phosphorylation of C-terminal sites proximal to CCR9 attenuates activation of G proteins

**Figure 5:**
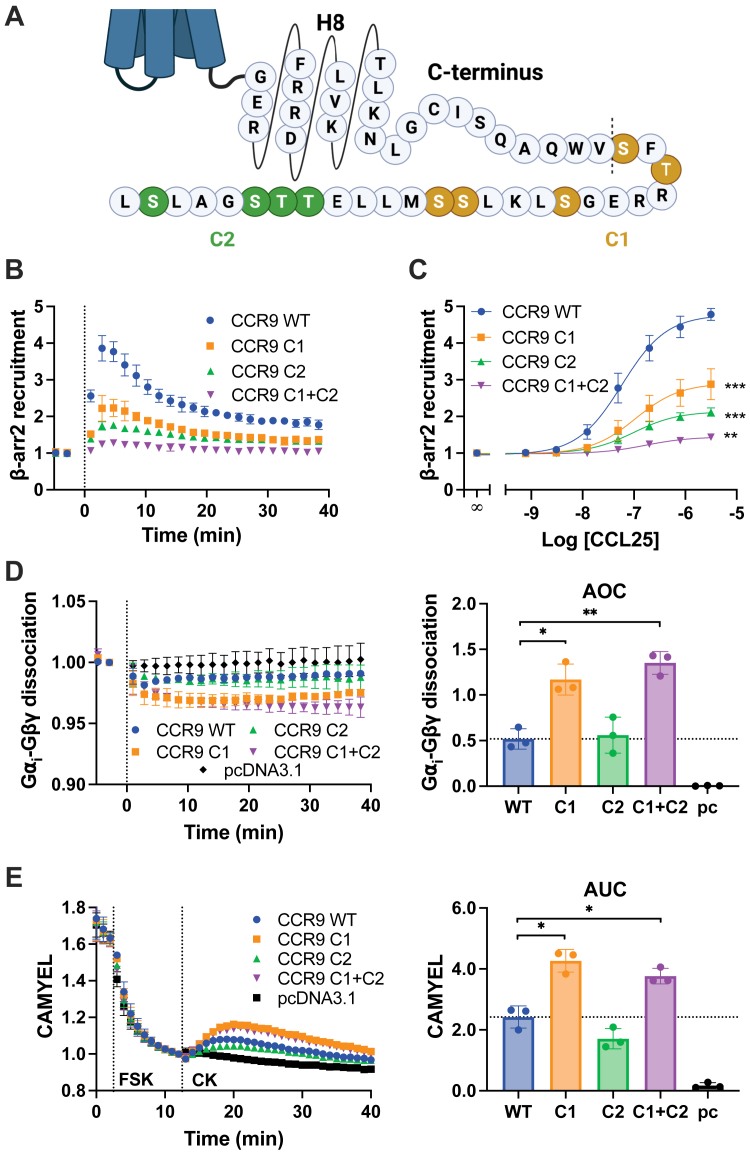
**Proximal phosphorylation sites regulate G protein activation.***A,* schematic view of CCR9 C-terminal mutants. Highlighted amino acids were mutated into alanines to form C1 (*yellow*) or C2 (*green*). CCL25-induced recruitment of GFP10-βarr2 toward CCR9-RlucII detected by BRET over time following stimulation with 200 nM chemokine (*B*) or across a titration of CCL25 concentrations (*C*) in HEK293 cells. *D,* activation of G_i_ proteins by CCR9 WT or ST/A mutants after stimulation by 100 nM CCL25 tracked by a decrease in BRET between G⍺_i_-Nluc and Gβγ-smV (*left*) and quantified by AOC analysis (*right*). WT data are repeated from Figure 1*B* for comparison. *E,* inhibition of forskolin-promoted cAMP production following stimulation of CCR9 ST/A mutants by 100 nM CCL25 in HEK293T using the BRET-based cAMP sensor CAMYEL (*left*) and quantified by AUC following chemokine stimulation (*right*). WT data are repeated from Figure 1*C* for comparison. Values represent the mean ± SD of three independent experiments performed in triplicate. Statistical significance was determined by one-way Brown–Forsythe and Welch ANOVA followed by a Dunnett's T3 multiple comparisons test (AOC) or using the extra sum-of-squares *F* test (DRC). ∗*p* < 0.05, ∗∗*p* < 0.001, and ∗∗∗*p* < 0.0001. AOC, area over the dissociation curve; AUC, area under the curve; BRET, bioluminescence resonance energy transfer; CCR9, C–C chemokine receptor type 9; DRC, dose–response curve; HEK293, human embryonic kidney 293 cell line.

GRK phosphorylation of GPCR C termini can produce specific modification patterns, which are proposed to coordinate specific responses *via* arrestin (43). The clear involvement of GRKs in suppressing CCR9 signaling presents the possibility that this regulation may be due to phosphorylation “barcodes” on the receptor C terminus. Phosphorylation of several sites (S345 and S368) on the CCR9 C terminus has been confirmed by mass spectrometry (44). To resolve the specific sites responsible for signal attenuation, two clusters of serines and threonines on the CCR9 C terminus (C1, S335A/T347A/S352A/S356A/S357A and C2, T362A/T363A/S364A/S368A) were mutated to alanines to selectively prevent GRK phosphorylation (Fig. 5*A*). The loss of phosphorylation was validated by β-arrestin2 recruitment measured by BRET. Both CCR9 C1 and CCR9 C2 showed impaired β-arrestin2 recruitment, with the latter having a slightly larger effect (Fig. 5, *B* and *C*). Indicating that both the proximal and the terminal phosphorylation sites are involved in the recruitment of arrestins. As expected, mutation of all the phosphorylation sites on the C-tail (C1 + C2) almost completely eliminated the CCL25-induced arrestin recruitment. Next, we tested G_i_ dissociation by the CCR9 ST/A mutants. Both CCR9 C1 and C1 + C2 showed a ∼two-fold increase in G protein dissociation (Fig. 5*D*), whereas C2 did not alter the rapid suppression of CCR9 signaling. An identical pattern was observed for suppression of cAMP production, with both CCR9 C1 and C1 + C2 showing decreased levels (Figs. 5*E*, S8). No correlation was observed between direct GRK recruitment toward the receptor and G protein coupling (Fig. S9). Signal attenuation is regulated by the proximal (C1), but not terminal (C2), phosphorylation sites, whereas GRK recruitment was similar for both constructs. This suggests that the enhancement observed for C1 and C1 + C2 is specifically because of changes in C-terminal phosphorylation and not recruitment of the GRKs. Mutations to CCR9 also had no effect on the basal G_i_ dissociation and cAMP levels (Fig. S10, *A and B*). Disambiguation of the C1 phosphorylation cluster with limited alanine substitutions was unable to resolve a further minimal inhibitory motif (Fig. S11), suggesting that the five positions of C1 all contribute to the muted G protein response of CCR9. Unexpectedly, eliminating phosphorylation of CCR9 by alanine substitution only produced ∼two-fold increase in G protein activation and not the ∼six-fold generated in the absence of GRKs or truncating the C terminus (Figs. 5*D* *versus* 2*B versus* 4*C*) and may suggest a role for the unmodified serine and threonine residues in coordinating G protein coupling. This discrepancy could not be explained by altered surface expression of the CCR9 mutants, as differences in the presented receptor levels did not correlate with enhancements to G protein activation (Fig. S12) nor did increasing the amount of transfected receptors increase the level of G protein signaling (Fig. S13).

### CCR9 internalization does not attenuate G protein signal attenuation

**Figure 6:**
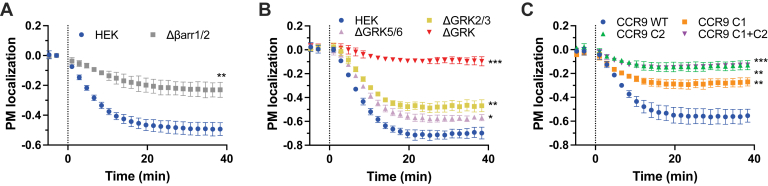
**Internalization not the driving factor for desensitization.***A,* CCL25-induced internalization measured as bystander BRET between CCR9-RlucII and rGFP-CAAX following stimulation with 200 nM chemokine in HEK293 parental and Δβarr1/2 cells (*A*), HEK293 parental, ΔGRK2/3, ΔGRK5/6, and ΔGRK2/3/5/6 (ΔGRK) cells (*B*), and of the CCR9 ST/A mutants in HEK293 cells (*C*). Values represent the mean ± SD of three independent experiments performed in triplicate. Statistical significance was determined by an unpaired *t* test, ∗*p* < 0.05, ∗∗*p* < 0.001, and ∗∗∗*p* < 0.0001. BRET, bioluminescence resonance energy transfer; CCR9, C–C chemokine receptor type 9; HEK293, human embryonic kidney 293 cell line.

GRK phosphorylation of GPCRs generally mediates receptor internalization through both arrestin-dependent and -independent mechanisms and is often considered a key step in receptor deactivation. Recent reports have shown that some receptors continue to signal from endosomal bodies (45); however, it is not known if this is the case for CCR9. Therefore, if CCR9 lacks intracellular signaling capabilities, phosphorylation-mediated internalization may account for the observed signal termination. Translocation of the receptor after ligand stimulation was monitored by bystander BRET between CCR9-RlucII and an acceptor anchored at the plasma membrane by a CAAX motif (rGFP-CAAX) (46). In WT cells, CCR9 shows robust and sustained translocation away from the plasma membrane with stimulation by CCL25 (Fig. 6*A*). In comparison, the level of CCR9 internalization in the Δβarr1/2 cells is significantly reduced to ∼50% that of WT cells. In contrast, CCL25-mediated CCR9 internalization is absent in ΔGRK2/3/5/6 cells but nearly WT-like with KO of GRK2/3 or GRK5/6 (Fig. 6*B*). These results would be consistent with CCR9 translocation being a driver of G protein signal attenuation, and only receptors on the surface are capable of coupling. However, internalization of the phosphosubstitution mutants reveals that trafficking of CCR9 is primarily mediated by the phosphorylation of the C2 sites (Fig. 6*C*). In the absence of these distal phosphorylation sites, CCR9 no longer shows agonist-mediated internalization. CCR9 C1 still internalizes with CCL25 stimulation albeit 50% that of WT. As loss of the C1 phosphorylation leads to enhanced CCR9 signaling and maintains partial internalization, the relocalization of CCR9 with CCL25 activation does not account for the acute signal suppression observed in Fig. 1.

### CCR9 phosphorylation disrupts interactions with G proteins

**Figure 7:**
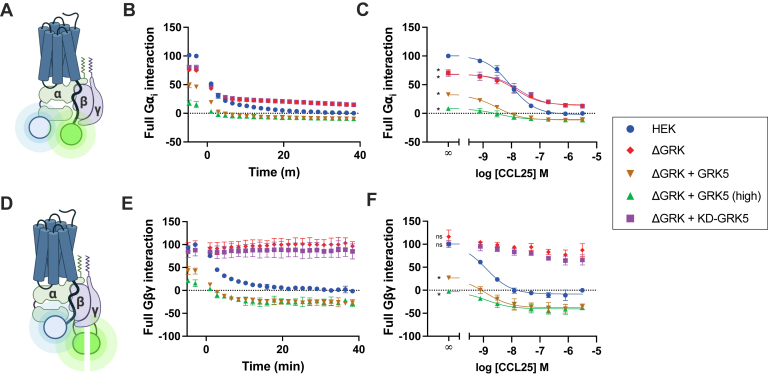
**Proximal phosphorylation impairs C-tail interaction with G⍺/Gβγ.***A,* schematic illustration of BRET pairs CCR9-mV and G⍺_i_-Nluc. *B,* change in interaction between G⍺_i_-Nluc and CCR9-mV following stimulation with 200 nM CCL25 at time 0 in HEK293 and HEK293 ΔGRK2/3/5/6 (ΔGRK) cells tracked by BRET (*B*) or across a titration of CCL25 concentrations (*C*). The GRK-deficient background was reconstituted by overexpression of GRK5, fourfold more GRK5 DNA, or KD-GRK5 as indicated. *D,* schematic overview of the BRET experiment to observe interaction between CCR9-RlucII and Gβγ-smV. *E,* CCL25-mediated change in interaction between Gβγ-smV and CCR9-RlucII in HEK293 and HEK293 ΔGRK cells observed by BRET following stimulation by 200 nM chemokine or (*F*) across a titration of CCL25 concentrations (*F*). As indicated, ΔGRK cells were cotransfected with GRK5, fourfold more GRK5 DNA, or KD-GRK5. Values represent the mean ± SD of three independent experiments performed in triplicate normalized to CCR9 WT response. Statistical significance using the extra sum-of-squares *F* test (*C*, *F*). ∗*p* < 0.0001. BRET, bioluminescence resonance energy transfer; CCR9, C–C chemokine receptor type 9; GRK, G protein–coupled receptor kinase; HEK293, human embryonic kidney 293 cell line.

Having eliminated the more common phosphorylation-mediated mechanisms for GPCR deactivation, we asked how might proximal phosphorylation inhibit G protein activation? One possible explanation is that the C-terminal phosphorylation sites are involved in stabilizing the CCR9–G protein ternary complex. Most structures of GPCRs and G proteins lack information on the receptor C terminus with a few notable exceptions modeling the proximal receptor C terminus in the cleft between the Gα and Gβ subunits (47). To resolve how phosphorylation impacts the CCR9–G protein complex formation, the interaction between the receptor and the effector was tracked by BRET. First, the effect of CCL25 activation on CCR9–Gα_i_ interactions was monitored between a receptor with a C-terminal mVenus acceptor and an Nluc on Gα_i_ (Fig. 7*A*). Full-length Gα_i_ was used for this assay instead of the mG_i_ from Fig. 1*A* to consider all interactions of the signaling heterotrimer. In WT cells, CCR9 shows rapid dissociation from G⍺_i_ with CCL25 stimulation (Fig. 7, *B* and *C*). The rate of dissociation outpaces the rate of internalization, suggesting that it is unlikely that the effect is the consequence of the receptor trafficking away from the heterotrimer (Fig. 7*B* *versus* Fig. 6). This suggests that G⍺_i_ precomplexes with CCR9, and receptor activation decreases this association. The CCL25-promoted dissociation between Gα_i_ and CCR9 is significantly reduced in ΔGRK2/3/5/6 cells where agonist-mediated phosphorylation is no longer possible. The remaining agonist-mediated decrease likely corresponds to activation of the G protein by the activated receptor and translocation of the Gα_i_ subunit away to initiate downstream signaling. The dissociative response of the full-length Gα_i_ with CCL25 stimulation conflicts with the association observed for mG_i_ (Fig. 1*A*). This is likely because of the engineered features of mG_i_, lack of lipidation, or the Gα_s_ scaffold, limiting potential receptor interactions compared with native Gα_i_ (48). To determine if phosphorylation is the driving factor of this dissociation, GRK5 and KD-GRK5 were cotransfected in the ΔGRK2/3/5/6 cells. Addition of GRK5 reduced basal Gα_i_–CCR9 precomplexes, whereas KD-GRK5 cotransfection matched the initial BRET level of ΔGRK2/3/5/6 cells alone. In both readdition conditions, GRK5 and KD-GRK5, the BRET between the receptor and G protein decreased with CCL25 treatment. Addition of KD-GRK5 matched the GRK-deficient background cells exactly, whereas GRK5 began lower and reached a similar BRET level as WT cells at the highest agonist concentrations. We suspected that the decrease in basal association between CCR9 and Gα_i_ with GRK5 addition was due to overexpression of the kinase increasing basal phosphorylation and any further decrease with stimulation was due to incomplete modification. Thus, we increased the amount of transfected GRK5 DNA (GRK5 high). As predicted, the higher expression condition lead to a further decrease in precomplexes and subsequently less further change with agonist stimulation, which is consistent with higher basal phosphorylation disrupting the interaction. Therefore, the dissociation of the Gα_i_–CCR9 precomplex appears to be regulated separately by two mechanisms, canonical receptor activation driving the G protein subunit to downstream signaling and GRK phosphorylation that disrupts productive coupling.

GPCR–G protein structures containing receptor C-terminal density suggest that interactions are also made with the Gβ subunit of the heterotrimer (47). The interaction between CCR9-RlucII and Gβγ-smV was tracked by BRET (Fig. 7*D*). Similar to Gα_i_, Gβγ quickly dissociated from CCR9 in WT cells following CCL25 stimulation (Fig. 7, *E* and *F*) at a faster rate than receptor internalization (Fig. 6). Unlike the partial effect observed for Gα_i_ in ΔGRK2/3/5/6 cells, the Gβγ:CCR9 BRET signal does not decrease with agonist stimulation. One explanation is that GRK2/3 act to translocate Gβγ in the WT cells, an interaction that would be absent in the cells lacking the GRKs. To address this, the same BRET experiment was performed in cells lacking either GRK2/3 or GRK5/6 (Fig. S14). Similar to G protein activation (Fig. S7), neither ΔGRK2/3 nor ΔGRK5/6 showed a deviation from the WT cell response, thereby confirming the lack of Gβγ dissociation is not because of the lack of Gβγ-interacting GRKs. To resolve if GRK phosphorylation or GRK interactions are mediating the Gβγ translocation, GRK5 and KD-GRK5 were cotransfected with the BRET components into ΔGRK2/3/5/6 cells. Like with Gα_i_, cotransfection with GRK5 reduced the basal CCR9-Gβγ BRET, likely because of increased background phosphorylation caused by GRK5 overexpression, and restored the CCL25-promoted dissociation observed in the WT cells (Fig. 7, *E* and *F*). Increasing GRK5 expression (GRK5 high) further reduced basal CCR9-Gβγ BRET as well as the CCL25-mediated response, similar as seen for G⍺_i_ (Fig. 7, *B* and *C*). The addition of KD-GRK5 had no effect on the CCR9-Gβγ BRET response with or without CCL25. This suggests that GRK phosphorylation of CCR9 leads to a destabilization of interactions with the Gβγ protein. Without phosphorylation, the Gβγ dimer remains associated with CCR9 during signal propagation, potentially acting to coordinate further G protein coupling.

## Discussion

**Figure 8:**
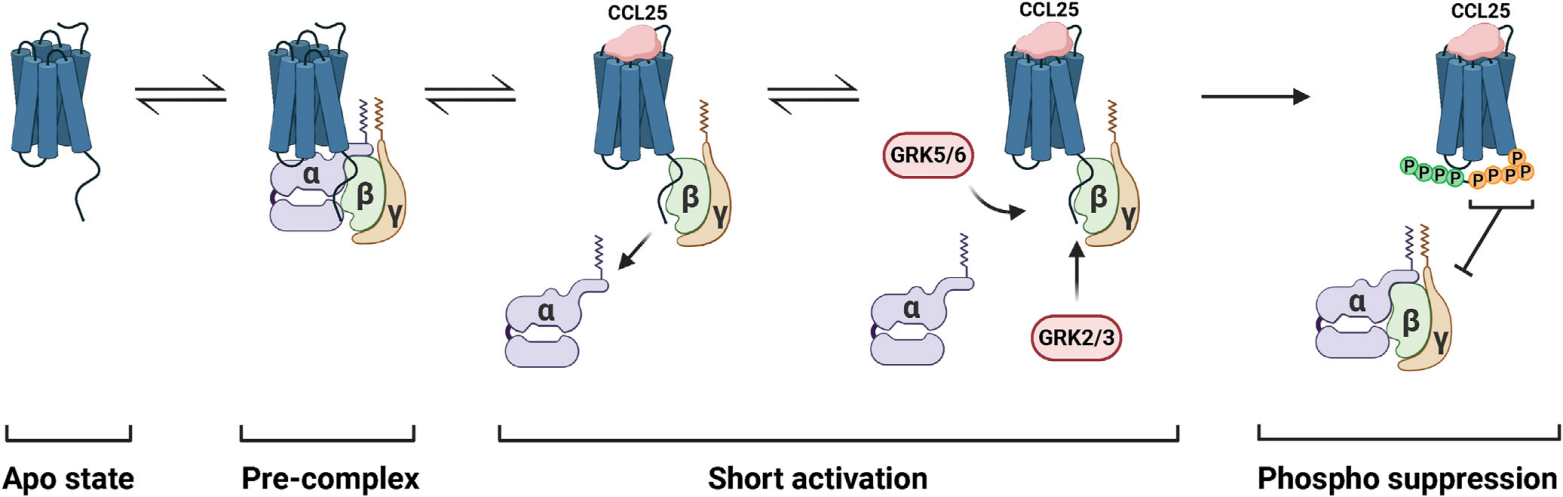
**CCR9 precomplexes with G proteins that is disturbed by proximal phosphorylation of the C-tail by GRKs upon receptor activation.** Empty CCR9 forms a precomplex with the full G protein heterotrimer (Fig. 7). Immediately upon chemokine binding, CCR9 is activated and initiates G protein signaling. The Gα unit dissociates to initiate downstream responses like cell migration, whereas the Gβγ remains associated with the activated receptor (Fig. 7, *B* and *E*). While activated, CCR9 is also primed for phosphorylation by GRKs. The kinases bind to the same interface as the G protein and compete for receptor interaction. GRK phosphorylation of the proximal receptor C terminus disrupts interactions and productive coupling of the G protein with CCR9. This leads to a dissociation of the CCR9–G protein complex (Fig. 7, *B* and *E*), rapidly terminates G protein activation, and allows for the reassociation of the heterotrimer (Fig. 1*B*). CCR9, C–C chemokine receptor type 9; GRK, G protein–coupled receptor kinase.

CCR9 mediates immune cell homeostasis in the gut, and overexpression or aberrant expression is tied to several inflammatory disorders and cancer metastasis. Therefore, suppressing receptor signaling is a promising therapeutical outcome, although direct antagonism of the receptor has proven difficult (15). Here, we describe a noncanonical mechanism of CCR9 signal termination, whereby the classical model of arrestin inhibition of GPCRs is circumvented. Instead, GRK-mediated phosphorylation of the CCR9 C terminus directly attenuates and suppresses G protein signaling. This allows for a limited signaling window during which G proteins and GRKs compete for access to the activated receptor (Fig. 1*B*). Recruitment of GRKs leads to phosphorylation, which inhibits further G protein signaling by interfering with productive coupling between the heterotrimer and the active GPCR (Fig. 7, *B* and *E*) and leads to rapid loss of G protein coupling in our HEK293 system (Fig. 8). Phosphorylation of specific sites mediates the observed attenuation, expanding the phosphorylation barcode theorem beyond arrestins. Alternate systems of GPCR regulation may present new avenues for specific therapeutic intervention.

The phosphorylation barcode hypothesis suggests that specific patterns of phosphorylation on a GPCR C terminus influence the interactions with effectors into specific outcomes. Although this is often discussed from an unbiased perspective, generally the only effect considered is the impact of the phosphorylation barcode on interactions and activation states of arrestins. An implicit assumption of these analyses is often that arrestins are the sole translators of different phosphorylation patterns into cellular responses (49). Here, we present evidence of arrestin-independent barcoding, where the phosphorylation alone is sufficient to terminate receptor signaling responses. Direct phosphoregulation of chemokine receptors is not a new concept. Efficient scavenging and internalization of the atypical chemokine receptor ACKR3 is dependent on GRK phosphorylation, whereas arrestins are dispensable (38, 50). In the case of ACKR3, the mechanisms connecting phosphorylation to functional regulation are unknown and may involve previously undescribed interactions. With CCR9, the addition of phosphates appears to destabilize the GPCR–G protein complex, which may prevent sustained responses. Phosphorylation of the C terminus may disrupt key interactions between the receptor and G protein through altering possible hydrogen bonding or changes to the electrostatics of the flexible domain. Importantly, this deactivation model does not require additional unknown interactors and can be fully explained by proposed direct contacts between the receptor and G protein. Though other factors may contribute, we prefer to propose the simplest possible model where phosphorylation of specific serine and threonine residues within the C terminus of CCR9 interferes with further G protein coupling, thereby terminating G protein signaling.

Canonically, the C terminus of GPCRs coordinates signal attenuation and termination by scaffolding arrestin to sterically impair G protein coupling. Recent studies have shown that the C termini of other GPCRs, including the prototypical GPCRs rhodopsin (51, 52) and β_2_-adrenergic receptor (30), interact with the cytoplasmic face of the receptor and competes with G protein interactions. Truncation of the C terminus led not only to a large increase in stimulated G protein signaling but also enhanced constitutive, unliganded G protein coupling. Structural and biophysical approaches have resolved interactions of the C-terminal tail with the receptor cytoplasmic face that are relieved upon receptor activation (30, 51). In the case of CCR9, interactions between the C terminus and the receptor are unknown. The tail is slightly positively charged in its basal state and shifts to negative when fully phosphorylated, which could interact with the generally positive electrostatics of the CCR9 cytoplasmic face. No change in constitutive activation is observed with ΔCT (Fig. S4), suggesting the CCR9 C terminus lacks an autoinhibitory role and likely does not make interactions with the receptor in the basal state. Curiously, the signal increase observed for other C-terminally truncated receptors was in the absence of arrestins (30). This indicates that a noncanonical arrestin-independent mechanism contributes to signal suppression in these other canonical GPCRs and may have been overlooked because of the dominance of the current paradigm of arrestin-mediated termination. In contrast, the rapid loss of CCR9 signaling is completely arrestin independent and only driven by the receptor C terminus and subsequent modifications. This suggests there may be degrees of relative contribution of arrestin-dependent and -independent signal termination regulating GPCR signaling, with CCR9 being an extreme example of arrestin independence.

How C-terminal phosphorylation lead to diminished G protein interactions and coupling is an open question. Despite an abundance of structures of GPCR–G protein complexes being reported, only a handful resolve any density for the receptor C terminus. In these few structures, the C terminus lays between the Gα and Gβ subunits of the G protein, forming contacts with both subunits (47, 53). In the structure of rhodopsin with its native G protein transducin, the residues that form these interactions include the phosphorylation sites S334 and T336 on the receptor, which contact C271, D290, and D291 on Gβ. When these sites are mutated to alanines, rhodopsin shows greater and more sustained G protein coupling (54). A similar effect is seen for CCR9, wherein substitution of the proximal phosphorylation sites on the C-tail relieves suppression of effective G protein coupling. Phosphorylation adds a negative charge to these residues, which may conflict with the electronegative aspartates forming the Gβ interface. Precomplexing has been reported for the muscarinic M1 and M3 receptors that are coordinated by a polybasic motif in the proximal C terminus (53, 55). CCR9 contains a miniature version of this sequence composed of the residues R348/R349/K354 within the C1 phosphorylation cluster. Phosphorylation of the C1 positions would dramatically change the electrostatics of the receptor tail, which may contribute to the loss of stable interactions. Preventing phosphorylation leads to not only greater and sustained G protein activation but also stabilizes the interactions between CCR9 and Gβγ (Fig. 7*E*). The stabilized interaction of Gβγ with CCR9 may act as an additional interface to recruit effector proteins such as exhausted Gα subunits, which would lead to reactivation of the heterotrimer, or GRK2/3 to promote the desensitization by the phosphorylation mechanism described here. CCR9 is readily regulated by GRK2/3 (Figs. 4, S7), suggesting that the associated Gβγ is not precluded from interacting with effectors. Thus, the tethering of Gβγ to CCR9 may contribute to the formation of localized signalosomes and restrain the migration signaling in the direction of CCL25 detection.

Increased stabilization of the CCR9–G protein complex may contribute to the enhanced G protein activation. Preassociation between the receptor and G protein would improve the probability of agonist stimulation leading to productive G protein coupling. Since submission of this article, precomplexing of several chemokine receptors has been documented, suggesting this may be a common mechanism for GPCRs driving migration signaling (56). The results with CCR9 presented here describe a mechanism for regulating the separation of these complexes, which is directly mediated by GRK phosphorylation. This does not preclude alternative mechanisms that may also contribute, such as phosphorylation by secondary-messenger–dependent kinases, and further investigation is needed to fully characterize the precomplexing interaction.

How might the muted CCR9 signaling translate to a physiological context? The HEK293 overexpression system used here is naturally far removed from the native immune cell environment. It is well established that CCR9 coupling to G proteins drives downstream signaling and cell migration; however, neither activation at the level of the G protein nor how the signal is terminated have been extensively investigated (22, 23, 24, 57). In our WT cell background, only limited signaling is observed, which is quickly suppressed by phosphorylation. However, this transient G protein activation may be sufficient for CCL25-driven cell migration and the rapid attenuation may have evolved as a tighter regulation mechanism. One could draw comparisons to rhodopsin, where receptor activation and deactivation is tightly controlled to improve sensitivity (58). By limiting the magnitude and length of signaling, CCR9 could provide specific and detailed positioning information for cells in the thymus and gut. Alternatively, the native system of CCR9 may have different kinase availabilities for phosphorylation. GRK levels in immune cells have been shown to be mediated by cytokine treatment, which in turn alters the localization of CCR9 proteins (59). Thus, this mechanism would allow for dynamic and cell-specific control over CCR9 signaling.

In conclusion, CCR9 activation is followed by rapid signal attenuation because of specific phosphorylation on the receptor C terminus. In contrast to canonical GPCR mechanisms, the phosphoregulation of CCR9 is not coordinated by arrestins sterically blocking G protein coupling. Instead, phosphorylation directly prevents productive G protein coupling. This noncanonical mechanism for signal termination may complicate efforts to drug the receptor. Other GPCRs likely display similar mechanisms but are often overlooked or missed because of dominant arrestin contributions. Thus, deactivation of GPCRs is perhaps more complicated than the canonical picture and may confound efforts to antagonize these receptors.

## Experimental procedures

### Materials

Unless stated otherwise, all chemicals and reagents were purchased from Melford or Sigma–Aldrich. HEK293T cells were obtained from American Type Culture Collection. HEK293 Δβ-arrestin1/2 cells (HEK293 Δβarr1/2) and corresponding parental cell lines were kindly gifted from Asuka Inoue (Tohoku University) (36). HEK293 ΔGRK2/3, ΔGRK5/6, ΔGRK2/3/5/6, and corresponding parental cell lines were generously provided by Carsten Hoffmann (Friedrich-Schiller-Universität Jena) (40).

### DNA constructs and site-directed mutagenesis

Human CCR9 (1-369), ACKR4 (1-350), and CXCR4 (1-352) were cloned into pcDNA3.1 expression vector either alone or followed by a C-terminal *Renilla* luciferase II (RlucII) or mVenus (mV). HA-H1R-Rluc8 (60), β-arrestin2-GFP10 (a gift from N. Heveker, Université de Montréal) (38), G⍺_i,3_ (61), G⍺_i_-Nluc, G⍺_q_-Nluc, GRK2-Nluc, GRK3-Nluc, GRK5-Nluc, GRK6-Nluc (gifts from D. Legler, Biotechnology Institute Thurgau) (26), split-mV(156-239)-Gβ_1_ (62), split-mV(1-155)-Gγ_2_ (gifts from N. Lambert, Augusta University) (62), NES-Venus-mGs/i143 (mG_i_) (25, 63), NES-Venus-mGs/q71 (25), mCitrine-EPAC-Rluc (CAMYEL) (28, 61), Nluc-C1B (29), mV-CAAX, and rGFP-CAAX (a gift from M. Bouvier, Université de Montréal) (46), and GRK5 and GRK5-K215R (KD-GRK5) (gifts from C. Hoffmann, Friedrich-Schiller-Universität Jena) (40) were described previously. Site-directed mutagenesis was performed using Q5 Site-Directed Mutagenesis Kit (New England Biolabs) and confirmed by Sanger sequencing.

### Chemokine purification from *Escherichia coli*

The chemokines CCL25 and CXCL12 were expressed and purified from *E. coli* as previously described (38, 64). Briefly, chemokine sequences were cloned into a pET21 vector together with N-terminal 8His-tag and enterokinase cleavage site. The generated complementary DNA was transformed into BL21(DE3)pLysS cells, and expression was controlled by IPTG induction. Inclusion bodies containing the chemokine were collected by sonication and dissolved in 50 mM Tris, 6 M guanidine–HCl, 50 mM NaCl, pH 8.0. The chemokine was purified using a nickel–nitrilotriacetic acid column, washed with 50 mM Mes, 6 M guanidine–HCl, 50 mM NaCl, pH 6.0 and eluted with 50 mM acetate, 6 M guanidine–HCl, 50 mM NaCl, pH 4.0. Purified chemokine was denatured with 4 mM DTT and later refolded in 50 mM Tris, 500 mM arginine–HCl, 1 mM EDTA, 1 mM GSSG, pH 7.5. Refolded chemokine was dialyzed in 20 mM Tris (pH 8.0) and 150 mM NaCl. Cleavage of the 8His-tag was done by addition of Enterokinase (New England Biolabs) and confirmed by SDS-PAGE and LC–MS. The cleaved material was polished on a nickel–nitrilotriacetic acid column, washed with 50 mM Tris (pH 8.0), and eluted in 6 M guanidine, 50 mM Mes, pH 6.0 and 6 M guanidine, 50 mM acetate, pH 4.0 as two separate fractions. The material was then bound to a C18 HPLC column (Gemini C18 110A; Phenomenex) (buffer A: 0.1% TFA, buffer B: acetonitrile + 0.1% TFA) and eluted by a linear gradient of buffer B (5–95%). The chemokines were collected, lyophilized, and stored at −80 °C.

### Transfection of HEK293 cells in suspension

Cells were cultured in Dulbecco's modified Eagle's medium (Thermo Fisher Scientific) supplemented with 1% penicillin and streptomycin (Gibco) and 10% fetal bovine serum (Bodinco) and maintained at 5% CO_2_ and 37 °C in a humidified atmosphere. HEK293/T cells were transfected with a total of 2 μg DNA per 1 × 10^6^ cells using 6 μg polyethyleneimine (PEI; Polysciences, Inc) as a transfection reagent in 150 mM NaCl. The DNA–PEI mixture was mixed and incubated for 15 min at room temperature. HEK293/T cells were detached using trypsin–EDTA solution (Thermo Fisher Scientific) and resuspended in Dulbecco's modified Eagle's medium. Cells were counted and subsequently added to the DNA–PEI mixture. Cells were seeded at 30 k/well in a white 96-well plate (Greiner) and incubated for 48 h unless stated otherwise. Cells were regularly tested for mycoplasma contamination.

### Mini-G**⍺** recruitment by BRET

HEK293 cells were transfected with 50 ng CCR9-RlucII, ACKR4-RlucII, HA-CXCR4-RlucII, or HA-H1R-Rluc8 and 250 ng NES-Venus-mG_s/i_143 or NES-Venus-mG_s/q_71 up to an amount of 2 μg with pcDNA3.1 as described previously. After 48 h incubation, cells were washed once with PBS and maintained in Hank's balanced salt solution (HBSS) supplemented with 0.1% bovine serum albumin (BSA; Fraction V; PanReac AppliChem). Cells were incubated with 5 μM of coelenterazine-h (CTZ-h; Promega) for 5 min before two baseline measurements were taken. Next, the cells were stimulated with 100 nM CCL25, CXCL12, or 10 μM histamine. Bioluminescence was measured using 475-30 nm and 535-30 nm filters on a PHERAstar plate reader for 1 h at 37 °C. BRET values were determined as the ratio of the red and blue luminescence readings (535-30/475-30). Results from three independent experiments were normalized to mock condition (no chemokine).

### G**⍺**–G**βγ** dissociation by BRET

HEK293, HEK293 Δβarr1/2, HEK293 ΔGRK2/3, HEK293 ΔGRK5/6, or HEK293 ΔGRK2/3/5/6 cells were transfected with 100 ng untagged receptor (or increasing amounts as indicated in the CCR9 DNA titration), 100 ng G⍺_i_-Nluc or G⍺_q_-Nluc, 500 ng Gβ-smV, 500 ng Gγ-smV, and 250 ng GRK5 or GRK5-K215R as indicated, up to an amount of 2 μg with pcDNA3.1 as described previously. After 48 h incubation, cells were washed once with PBS and maintained in HBSS supplemented with 0.1% BSA. Cells were incubated with 2.5 μM of furimazine (Promega) for 5 min before two prestimulation measurements were taken. Chemokine addition, measurements, and data analysis were performed as described previously (see Mini-G⍺ recruitment by BRET section). Results from three independent experiments were normalized to the mock-treated condition (no chemokine). Quantification is done by integration of the area over the BRET curves (GraphPad Prism; GraphPad Software, Inc).

### cAMP measurements by BRET

HEK293/T cells were transfected with 400 ng untagged receptor, 800 ng CAMYEL, and 400 ng G⍺_i,3_ up to an amount of 2 μg with pcDNA3.1 as described previously. After 48 h incubation, cells were washed once with PBS and maintained in HBSS supplemented with 0.1% BSA. Cells were incubated with 5 μM of CTZ-h (Promega) for 5 min before three baseline measurements were taken. cAMP levels were amplified with 10 μM FSK (Sigma–Aldrich) and measured for 10 min. Next, the cells were stimulated with 100 nM CCL25 or CXCL12, and bioluminescence was measured as described before (see Mini-G⍺ recruitment by BRET section). Results from three independent experiments were normalized to baseline to correct for basal differences.

### C1B recruitment by BRET

HEK293/T cells were transfected with 100 ng untagged receptor, 50 ng Nluc-C1B, and 500 ng mV-CAAX up to an amount of 2 μg with pcDNA3.1 as described previously. Stimulations, measurements, and data analysis were performed as described previously (see G⍺–Gβγ dissociation by BRET section).

### **β**-arrestin2 recruitment by BRET

HEK293, HEK293 ΔGRK2/3, HEK293 ΔGRK5/6, or HEK293 ΔGRK2/3/5/6 cells were transfected with 50 ng CCR9-RlucII and 1 μg GFP10-β-arrestin2 up to an amount of 2 μg with pcDNA3.1 as described previously. After 48 h incubation, cells were washed once with PBS and maintained in HBSS supplemented with 0.1% BSA. Cells were incubated with 5 μM of Prolume Purple (Prolume Ltd) for 5 min before three baseline measurements were taken. Next, the cells were stimulated with increasing concentrations of CCL25. Bioluminescence was measured at 410-80 nm and 515-30 nm using a PHERAstar plate reader for 1 h at 37 °C. BRET values were determined as the ratio of the red and blue luminescence readings (515-30/410-80). Results from three independent experiments were normalized to mock (no chemokine).

### Agonist-mediated internalization assay by BRET

HEK293, HEK293 Δβarr1/2, HEK293 ΔGRK2/3, HEK293 ΔGRK5/6, or HEK293 ΔGRK2/3/5/6 cells were transfected with 50 ng CCR9-RlucII and 200 ng rGFP-CAAX up to an amount of 2 μg with pcDNA3.1 as described previously. Stimulations, measurements, and data analysis were performed as described previously (see β-arrestin2 recruitment by BRET section). Results from three independent experiments were normalized to WT response as indicated, and ΔBRET was calculated as BRET(CCL25)-BRET(mock).

### Full Gα_i_ and G**βγ** interaction by BRET

HEK293, HEK293 ΔGRK2/3, HEK293 ΔGRK5/6, or HEK293 ΔGRK2/3/5/6 cells were transfected with 250 ng CCR9-mV and 50 ng G⍺_i_-Nluc or 50 ng CCR9-RlucII and 250 ng Gβ-smV, and 250 ng Gγ-smV, with additional 250 ng of GRK5 or GRK5-K215R (1000 ng for GRK5 [high]) as indicated, up to an amount of 2 μg with pcDNA3.1 as described previously. After 48 h incubation, cells were washed once with PBS and maintained in HBSS supplemented with 0.1% BSA. Cells were incubated with 2.5 μM of furimazine (G⍺_i_ assay) or 5 μM CTZ-h (Gβγ assay) for 5 min before two baseline measurements were taken. Next, the cells were stimulated with increasing concentrations of CCL25. Measurements were performed as described previously (see Mini-G⍺ recruitment by BRET section). Results from three independent experiments were normalized to WT response.

### Direct GRK recruitment by BRET

HEK293 cells were transfected with 50 ng GRK-Nluc and 250 ng CCR9-mV up to an amount of 2 μg with pcDNA3.1 as described previously. Stimulations, measurements, and data analysis were performed as described previously (see G⍺–Gβγ dissociation by BRET section).

### Surface expression by flow cytometry

HEK293, HEK293 ΔGRK2/3, HEK293 ΔGRK5/6, or HEK293 ΔGRK2/3/5/6 cells were transfected with 100 ng CCR9 and up to an amount of 2 μg with pcDNA3.1 as previously described. After 48 h incubation, cells were washed once with PBS and transferred to a 96-well conical plate (Guava compatible 96-well plate; Greiner) using Accutase (Thermo Fisher Scientific). The plates were held at 4 °C and centrifuged at 350*g* for 3 min between each step. Cells were washed twice with cold FACS buffer (filtered PBS + 0.5% BSA). Next, cells were incubated with anti-hCCR9A antibody (R&D Systems; MAB1791) for 1 h at 4 °C. After incubation, cells were washed twice with cold FACS buffer and consequently incubated with anti-mouse F(ab)2 IgG (H + L) PE-conjugated antibody (R&D Systems; F0102B) for 1 h in the dark at 4 °C. Cells were washed three times with cold FACS buffer and finally resuspended in FACS buffer. Mean yellow fluorescence was measured on the Guava easyCyte (Cytek) and normalized to WT and pcDNA3.1 values.

### Statistical analysis

Statistical analyses were performed using GraphPad Prism 10. Bar and symbol representation, along with error bars, are described in figure legends. Scatter plots show the mean of three independent experiments, each measured in triplicate. For bar charts, the bars represent the mean of three independent experiments, whereas the points depict the mean values of the individual experiments measured in triplicate. All errors are reported as SD. The dose–response curves were plotted using a sigmoidal dose–response model (log[agonist] *versus* response, three parameters) using GraphPad Prism 10.

$$y=\text{Bottom}\;\frac{\left(Top-Bottom\right)}{1+10^{LogEC50-x}}$$

The AOC or area under the curve was determined in GraphPad Prism 10 (baseline = 1.0). Statistical significance of dose–response curves was determined using the extra sum-of-squares *F* test. For multiple comparisons, one-way Brown–Forsythe and Welch ANOVA followed by a Dunnett's T3 multiple comparisons test was applied. All other statistical comparisons were done by an unpaired *t* test.

## Data availability

All data for these studies are contained within this article.

## Supporting information

This article contains supporting information.

## Conflict of interest

The authors declare that they have no conflicts of interest with the contents of this article.
