# Supplementary material for: Signal termination of the chemokine receptor CCR9 is governed by an arrestin-independent phosphorylation mechanism
Source: J Biol Chem. 2025 Mar 26;301(5):108462. doi: 10.1016/j.jbc.2025.108462 (PMC12147180; doi:10.1016/j.jbc.2025.108462)
Supplement: Figures S1–S14 and Table S15 [file mmc1.pdf]

Supporting information

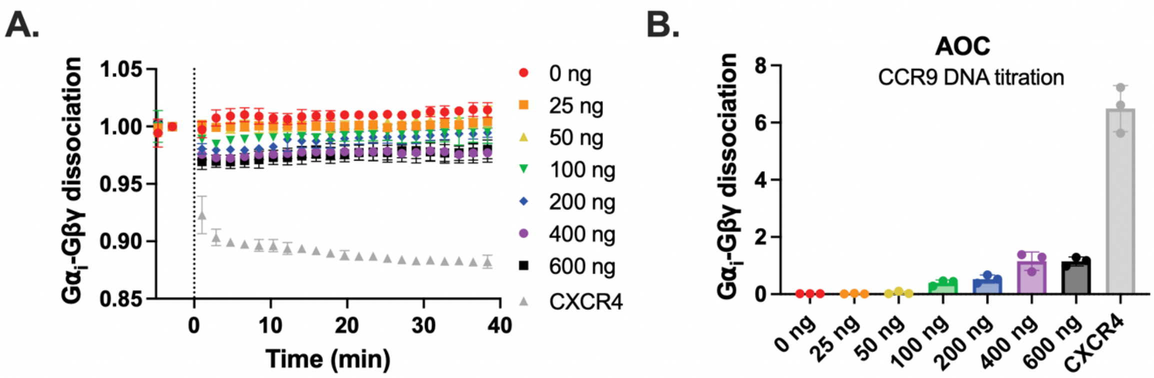

Figure S1. Increasing the amount of transfected CCR9 DNA slightly enhanced heterotrimer activation, albeit still less than CXCR4. (A) Ligand-induced activation of G_i proteins measured as dissociation of $G\alpha_i$-Nluc and Gβγ-split-mVenus (Gβγ-smV) in HEK293 cells across a titration of transfected CCR9 DNA. CXCR4 data is repeated from Fig.1B for comparison. (B) Quantification of $G\alpha_i$-Gβγ dissociation by integration of the area over the BRET curves (AOC). Values represent the mean ± SD of three independent experiments performed in triplicate.

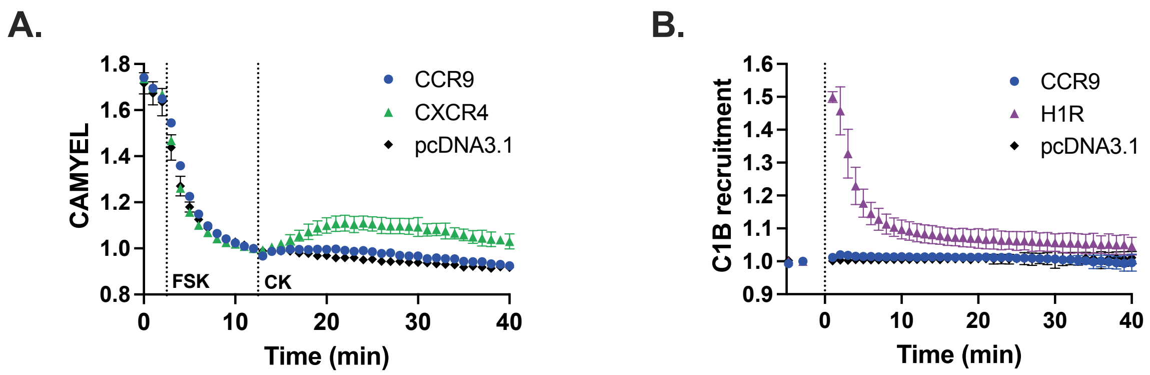

Figure S2. CCR9 shows limited downstream G_i and G_q signaling in HEK293 cells. (C) Inhibition of cAMP production by CCR9 and CXCR4 using the BRET-based cAMP sensor CAMYEL. Cells were treated with 10 µM forskolin (FSK) to stimulate cAMP production for 10 min, followed by 100 nM chemokine (CK, CCL25 or CXCL12). (B) Recruitment of C1B to the membrane measured as bystander BRET between Nluc-C1B and mV-CAAX by CCR9 and H1R following stimulation with 100 nM CCL25 or 10 µM histamine, respectively. Values represent the mean ± SD of three independent experiments performed in triplicate.

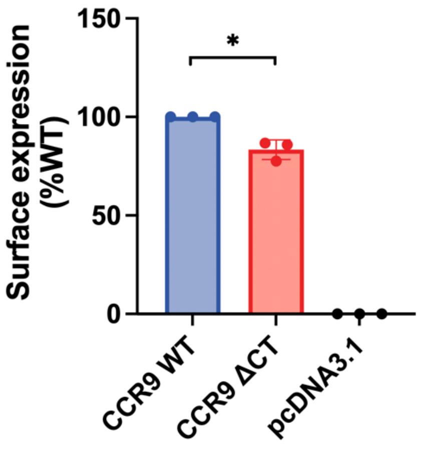

Figure S3. Surface expression of CCR9 ΔCT is slightly less than CCR9 WT. Surface expression of untagged CCR9 in transfected HEK293 cells measured by flow cytometry. Bars represent the mean ± SD of three independent experiments performed in triplicate. The average results of individual experiments are presented as points. Statistical significance was determined by a t-test. *P<0.05.

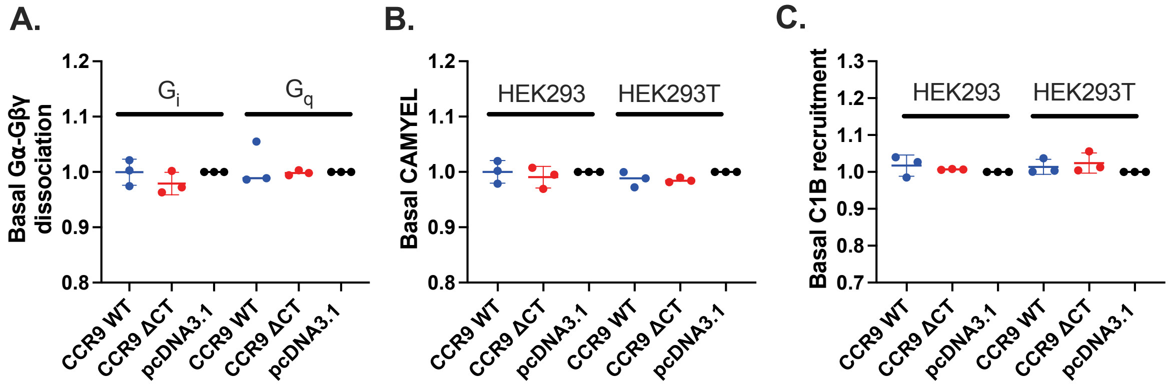

Figure S4. Neither WT nor ΔCT CCR9 show constitutive, agonist independent G protein activation. (A) Basal G_i and G_q activation measured as dissociation of $G\alpha_i$-Nluc or $G\alpha_q$-Nluc and Gβγ-smV in cells in the presence of CCR9 WT and CCR9 ΔCT. (B) Basal suppression of cAMP production by CCR9 WT and CCR9 ΔCT using the BRET-based cAMP sensor CAMYEL. (C) Basal C1B recruitment by CCR9 WT and CCR9 ΔCT measured as bystander BRET between Nluc-C1B and mV-CAAX. Values represent the mean ± SD of three independent experiments performed in triplicate. The average results of individual experiments are presented as points. No differences were statistically significant.

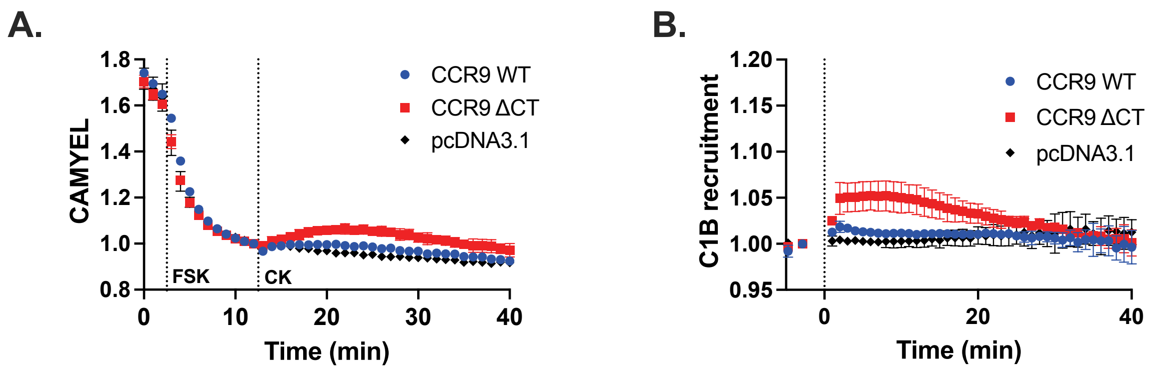

Figure S5. Truncating the CCR9 C-terminus improves CCL25-induced downstream G_i and G_q signaling in HEK293 cells. (A) Inhibition of cAMP production by CCR9 WT and CCR9 ΔCT using the BRET-based cAMP sensor CAMYEL. Cells were treated with 10 µM forskolin (FSK) to stimulate cAMP production for 10 min, followed by 100 nM chemokine (CK, CCL25). (B) Recruitment of C1B to the membrane measured as bystander BRET between Nluc-C1B and mV-CAAX by CCR9 WT and CCR9 ΔCT following stimulation with 100 nM CCL25. Values represent the mean ± SD of three independent experiments performed in triplicate.

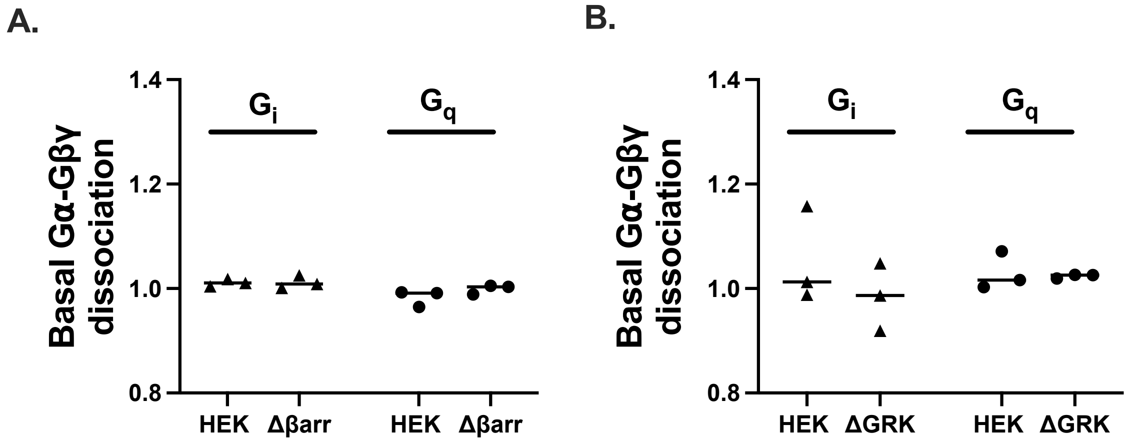

Figure S6. No change is observed in constitutive heterotrimer dissociation in Δβarr1/2 or ΔGRK cells. Basal G_i or G_q activation measured as dissociation of $G\alpha_{i/q}$-Nluc and Gβγ-smV in Δβarr1/2 (A), ΔGRK (B), and their corresponding parental HEK293 cell line in the presence of CCR9. BRET ratios are normalized to pcDNA3.1.

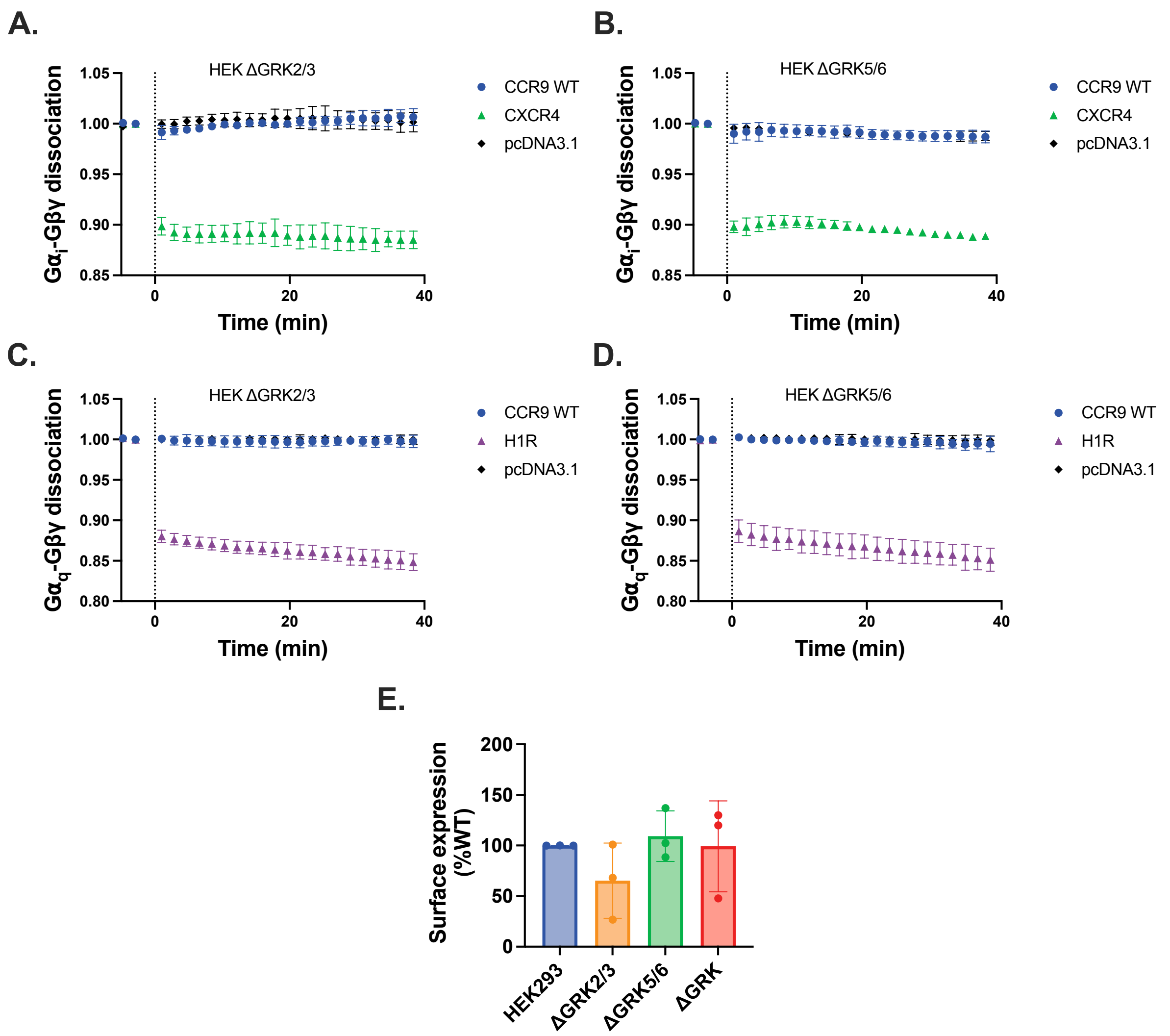

Figure S7. G protein activation by CCR9 is not enhanced in GRK2/3 or GRK5/6 knock-outs. Ligand-induced activation of G_i proteins measured as dissociation of $G\alpha_i$-Nluc and Gβγ-smV in HEK293 ΔGRK2/3 (A) and ΔGRK5/6 (B) cells by CCR9 and CXCR4 upon stimulation with 100 nM CCL25 or CXCL12, respectively. Ligand-induced activation of G_q proteins measured as dissociation of $G\alpha_q$-Nluc and Gβγ-smV in HEK293 ΔGRK2/3 (C) and ΔGRK5/6 (D) cells by CCR9 and H1R upon stimulation with 100 nM CCL25 or 10 μM histamine, respectively. Values represent the mean ±SD of three independent experiments performed in triplicate. (E) Surface expression of untagged CCR9 in transfected HEK293, ΔGRK2/3, ΔGRK5/6, and ΔGRK cells measured by flow cytometry. Bars represent the mean ± SD of three independent experiments performed in triplicate. The average results of individual experiments are presented as points.

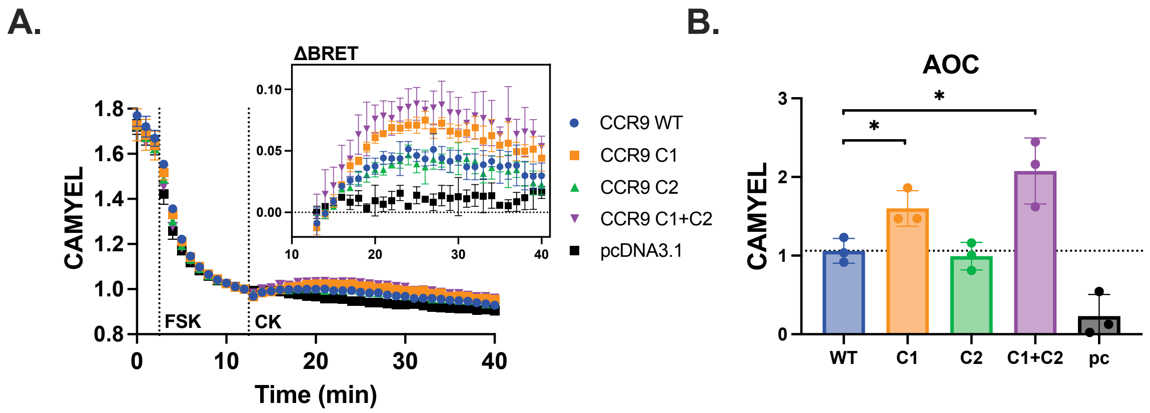

Figure S8. **Proximal phosphorylation sites suppress downstream G_i signaling in HEK293 cells.** (A) Inhibition of cAMP production by CCR9 using the BRET-based cAMP sensor CAMYEL. Cells were treated with 10 µM forskolin (FSK) to stimulate cAMP production for 10 min, followed by 100 nM chemokine (CK, CCL25). (B) Quantification of cAMP levels by integration of the area over the BRET curves. Values represent the mean ± SD of three independent experiments performed in triplicate. Statistical significance was determined by one-way Browns-Forsythe & Welch ANOVA followed by a Dunnett's T3 multiple comparisons test. *$P<0.05$.

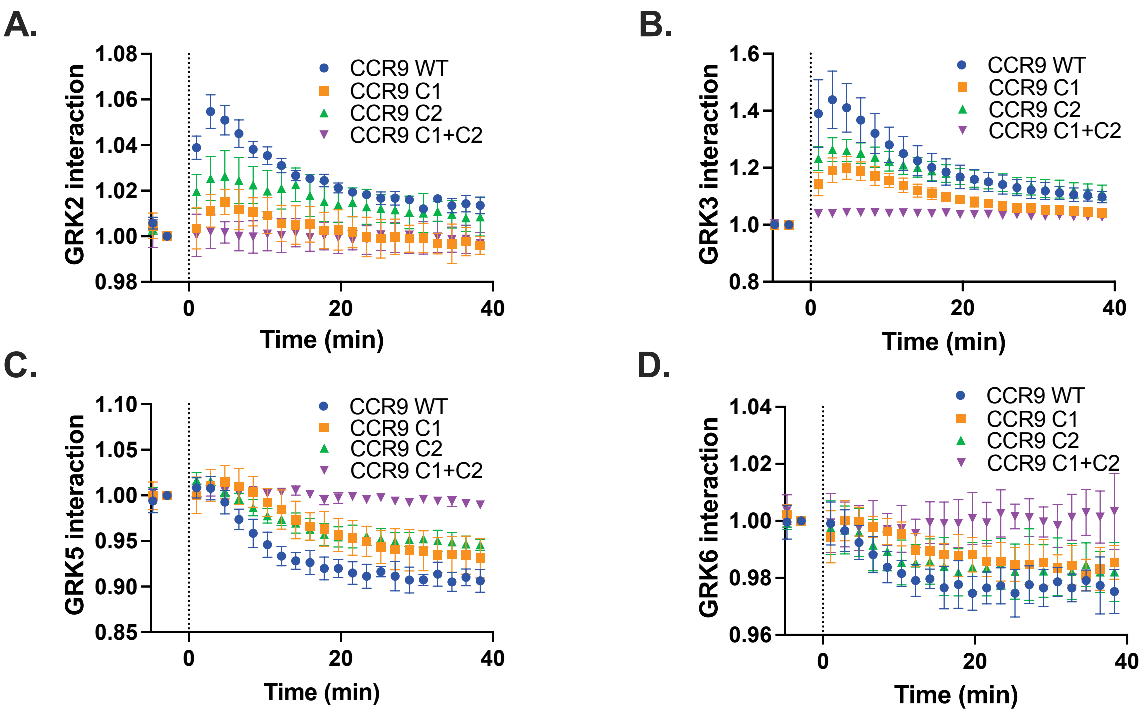

Figure S9. Enhanced G protein coupling by CCR9 ST/A constructs does not correlate with differences in GRK recruitment. Direct GRK recruitment of GRK2-Nluc (A), GRK3-Nluc (B), GRK5-Nluc (C), and GRK6-Nluc (D) towards CCR9-mVenus in HEK293 cells upon 200 nM CCL25 detected by BRET over time. Values represent the mean ± SD of three independent experiments performed in triplicate.

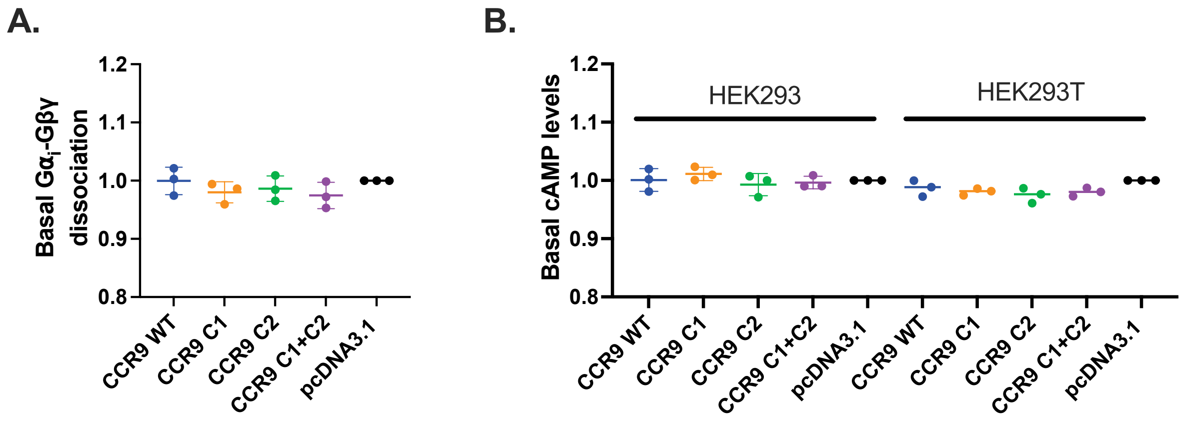

Figure S10. ST/A CCR9 constructs do not constitutively activate G proteins. (A) Basal G_i activation measured as dissociation of Gα$_i$-Nluc and Gβγ-smV in cells in the presence of CCR9 or pcDNA3.1. CCR9 WT data is repeated from Fig. S4 for comparison. (B) Basal suppression of cAMP production by CCR9 or pcDNA3.1 using the BRET-based cAMP sensor CAMYEL. Values represent the mean ± SD of three independent experiments performed in triplicate and normalized to pcDNA3.1 condition, while points represent the averages from the individual experiments.

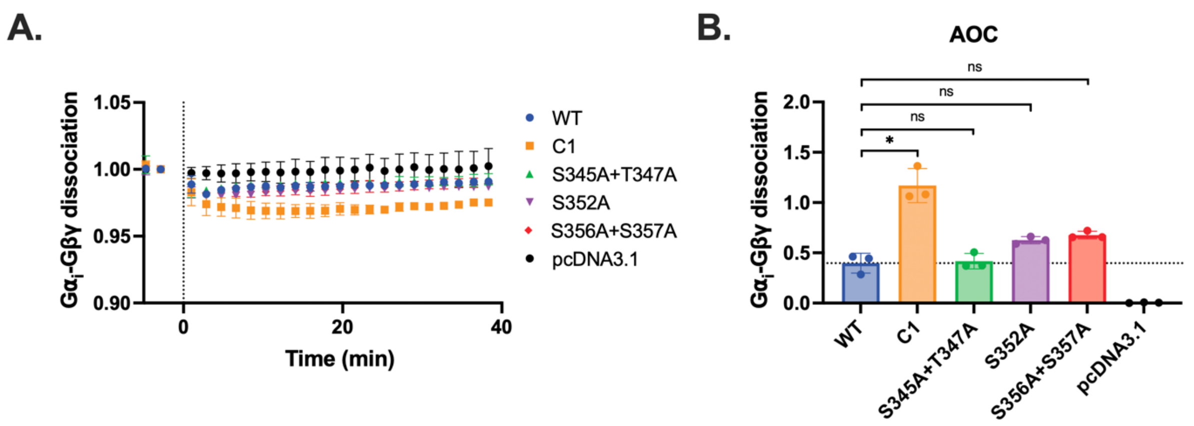

Figure S11. Disambiguation of the C1 phosphorylation cluster did not resolve the key residues for the observed enhanced Gα$_i$-Gβγ dissociation. (A) Ligand-induced activation of G$_i$ proteins measured as dissociation of Gα$_i$-Nluc and Gβγ-smV in HEK293 cells after stimulation of CCR9 WT and ST/A mutants with 100 nM chemokine. WT data is repeated from Fig. 1B and C1 from Fig. 5D for comparison. Values represent the mean ± SD of three independent experiments performed in triplicate. (B) Quantification of Gα$_i$-Gβγ dissociation by integration of the area over the BRET curves. Values represent the mean ± SD of three independent experiments performed in triplicate and normalized to pcDNA3.1 condition, while points represent the averages from the individual experiments. Statistical significance was determined by one-way Browns-Forsythe & Welch ANOVA followed by a Dunnett's T3 multiple comparisons test. *P<0.05.

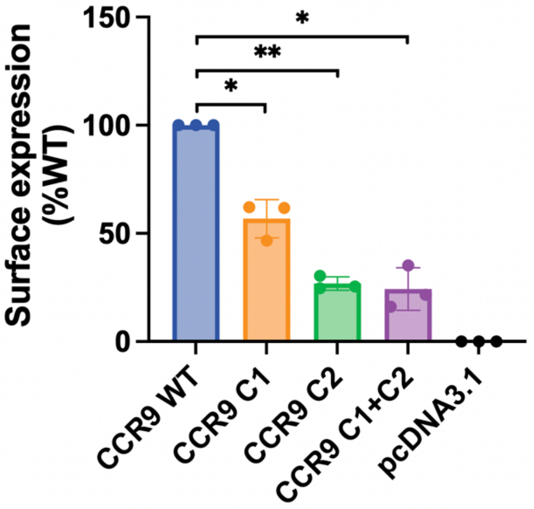

Figure S12. Surface expression of CCR9 WT and ST/A mutants determined by flow cytometry. Surface expression of untagged CCR9, CCR9 C1, CCR9 C2, and CCR9 C1+C2 in transfected HEK293 cells determined by flow cytometry. Values represent the mean ± SD of three independent experiments performed in triplicate. Points present the average from individual experiments. CCR9 WT data is repeated from Fig. S3 for comparison. Statistical significance was determined by one-way Browns-Forsythe & Welch ANOVA followed by a Dunnett's T3 multiple comparisons test. *P<0.05,**P<0.001.

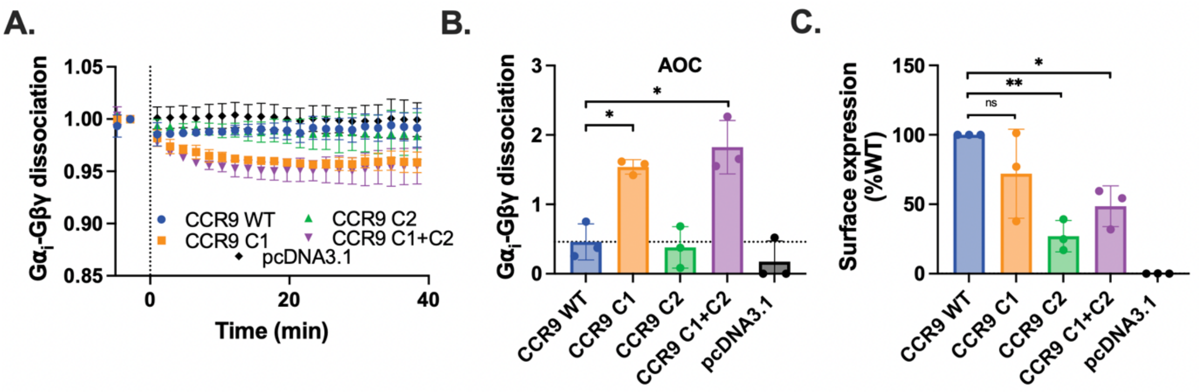

Figure S13. Attempts to increase surface expression of CCR9 mutants did not change effect on G protein coupling. To address the discrepancy in surface expression observed in Fig. S12, transfected DNA amounts were adjusted to attempt to match the surface expression of CCR9 WT (2xC1, 4xC2, 4xC1+C2). (A) Ligand-induced activation of G_i proteins measured as dissociation of $G\alpha_i$-Nluc and Gβγ-smV in HEK293 cells in the presence of CCR9 or empty pcDNA3.1 upon stimulation of 100 nM chemokine. (B) Quantification of $G\alpha_i$-Gβγ dissociation by integration of the area over the BRET curves. (C) Surface expression of untagged CCR9, CCR9 C1, CCR9 C2, CCR9 C1+C2 in transfected HEK293 cells quantified by flow cytometry. Values represent the mean ± SD of three independent experiments performed in triplicate. Points represent the average value from individual experiments. Statistical significance was determined by one-way Browns-Forsythe & Welch ANOVA followed by a Dunnett's T3 multiple comparisons test. *P<0.05,**P<0.001.

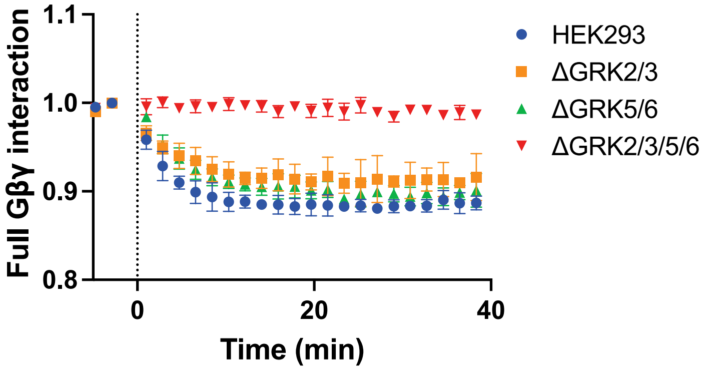

Figure S14. GRK2/3 do not sequester the Gβγ protein. Ligand-induced interaction between Gβγ-smV and CCR9-RlucII in HEK293, ΔGRK2/3, ΔGRK5/6, ΔGRK cells measured by BRET over time following stimulation with 200 nM chemokine. Values represent the mean ± SD of three independent experiments performed in triplicate normalized to mock condition (no chemokine).

Table S15 Statistics

Fig.	Condition	Emax		Log EC50		Statistical test
		P-value	Value ± SD	P-value	Value ± SD	
**2b**	CCR9 WT	N.A	0.40 ± 0.098	N.A.		t-test
	CCR9 ΔCT	0.0028	2.61 ± 0.29			
**2c**	CCR9 WT	N.A.	0.45 ± 0.29	N.A.		t-test
	CCR9 ΔCT	0.0078	1.98 ± 0.08			
**2d**	CCR9 WT	N.A.	2.42 ± 0.36	N.A.		t-test
	CCR9 ΔCT	<0.0001	7.58 ± 0.24			
	CXCR4	0.70* *Compared to ΔCT	7.66 ± 0.22			
**2e**	CCR9 WT	N.A.	0.74 ± 0.34	N.A.		t-test
	CCR9 ΔCT	0.017	2.42 ± 0.56			
**4b**	HEK P	N.A.	5.00 ± 0.33	N.A	-7.28 ± 0.10	Extra sum-of-squares F test
	ΔGRK2/3	<0.0001	3.51 ± 0.34	0.31	-7.19 ± 0.17	
	ΔGRK5/6	<0.0001	4.05 ± 0.04	0.58	-7.24 ± 0.13	
	ΔGRK	<0.0001	1.45 ± 0.16	0.59	-7.13 ± 0.42	
**4d**	HEK P	N.A	0.40 ± 0.10	N.A		Browns-Forsythe & Welch ANOVA
	HEK ΔGRK	0.0040	2.22 ± 0.27			
	HEK + KD-GRK5	0.087* *Compared to ΔGRK	1.58 ± 0.22			
**4f**	HEK P	N.A.	0.24 ± 0.02	N.A.		Browns-Forsythe & Welch ANOVA
	HEK ΔGRK	0.035	1.10 ± 0.19			
	HEK + KD-GRK5	0.19* *Compared to ΔGRK	0.79 ± 0.09			
**5c**	CCR9 WT	N.A	4.78 ± 0.16	N.A	-7.23 ± 0.13	Extra sum-of-squares F test
	CCR9 C1	<0.0001	2.88 ± 0.42	0.0701	-6.97 ± 0.28	

	CCR9 C2	<0.0001	2.12 ± 0.11	0.1757	-7,0 ± 0.15	
	CCR9 C1+C2	0.0043	1.43 ± 0.05	0.2591	-6.78 ± 0.20	
**5e**	CCR9 WT	N.A	0.52 ± 0.11	N.A		Browns-Forsythe & Welch ANOVA
	CCR9 C1	0.0331	1.17 ± 0.17			
	CCR9 C2	0.9945	0.56 ± 0.20			
	CCR9 C1+C2	0.0032	1.35 ± 0.12			
**5f**	CCR9 WT	N.A	2.42 ± 0.36	N.A		Browns-Forsythe & Welch ANOVA
	CCR9 C1	0.0115	4.27 ± 0.38			
	CCR9 C2	0.1967	1.71 ± 0.34			
	CCR9 C1+C2	0.0201	3.76 ± 0.26			
**6a**	HEK P	N.A	-0.49 ± 0.04	N.A		t-test
	Δβarr1/2	<0.0025	-0.23 ± 0.05			
**6b**	WT	N.A	-0.70 ± 0.05	N.A		t-test
	ΔGRK2/3	0.0049	-0.47 ± 0.05			
	ΔGRK5/6	0.0279	-0.57 ± 0.03			
	ΔGRK	0.0001	-0.10 ± 0.04			
**6c**	CCR9 WT	N.A	-0.55 ± 0.05	N.A		t-test
	CCR9 C1	0.0030	-0.27 ± 0.03			
	CCR9 C2	0.0007	-0.13 ± 0.04			
	CCR9 C1+C2	0.0041	-0.13 ± 0.01			

**7c**	HEK P	N.A	100	N.A	-8.03 ± 0.07	Extra sum-of-squares F test
	ΔGRK	<0.0001	71.62 ± 4.21	0.0295	-7.84 ± 0.17	
	ΔGRK + GRK5	<0.0001	32.17 ± 0.47	<0.0001	-8.5 ± 0.20	
	ΔGRK + GRK5 (high)	<0.0001	8.50 ± 3.31	0.050	-8.46 ± 42	
	ΔGRK + KD-GRK5	<0.0001	70.21 ± 5.76	0.0057	-7.73 ± 24	
**7f**	HEK P	N.A.	100	N.A.	-8.89 ± 0.11	Extra sum-of-squares F test
	ΔGRK	0.298	116.35 ± 14.5	N.A.	N.A.	
	ΔGRK + GRK5	<0.0001	27.08 ± 4.39	0.81	-8.92 ± 0.28	
	ΔGRK + GRK5 (high)	<0.0001	-2.70 ± 3.59	0.95	-8.88 ± 0.46	
	ΔGRK + KD-GRK5	0.9891	100.59 ± 7.70	N.A.	N.A.	
**S3**	CCR9 WT	N.A	N.A	N.A.		t-test
	CCR9 ΔCT	0,029	83,39 ± 5,00			
**S7**	HEK	N.A.	N.A.	N.A.		Browns-Forsythe & Welch ANOVA
	ΔGRK2/3	0.47	65.28 ± 37.15			
	ΔGRK5/6	0.89	109 ± 24.96			
	ΔGRK	0.99	99.21 ± 44.89			
**S8**	CCR9 WT	N.A.	1.06 ± 0.16	N.A.		Browns-Forsythe & Welch ANOVA
	CCR9 C1	0.031	1.60 ± 0.23			
	CCR9 C2	0.645	0.99 ± 0.18			
	CCR9 C1+C2	0.018	2.08 ± 0.42			

**S11**	WT	N.A.	0.40 ± 0.10	N.A.	Browns-Forsythe & Welch ANOVA
	C1	0.0214	1.17 ± 0.17		
	S345A + T347A	0.99	0.42 ± 0.08		
	S352A	0.1037	0.63 ± 0.04		
	S356A + S357A	0.0623	0.68 ± 0.04		
**S12**	CCR9 WT	N.A	N.A	N.A	Browns-Forsythe & Welch ANOVA
	CCR9 C1	0.036	56.83 ± 8.84		
	CCR9 C2	0.0015	26.83 ± 3.12		
	CCR9 C1+C2	0.0137	24.27 ± 9.84		
**S13b**	CCR9 WT	N.A.	0.46 ± 0.26	N.A.	Browns-Forsythe & Welch ANOVA
	CCR9 C1	0.0179	1.54 ± 0.10		
	CCR9 C2	0.9924	0.38 ± 0.3		
	CCR9 C1+C2	0.0222	1.82 ± 0.38		
**S13c**	CCR9 WT	N.A.	N.A.	N.A.	Browns-Forsythe & Welch ANOVA
	CCR9 C1	0.27	72.04 ± 32.04		
	CCR9 C2	0.0079	27.04 ± 11.30		
	CCR9 C1+C2	0.026	48.58 ± 14.67		
